# Inoculation of *Bacillus cabrialesii* modulates plant growth and wilt disease of chickpea cultivars pre-challenged with *Fusarium oxysporum* f. sp. *ciceris*

**DOI:** 10.1186/s12866-026-05357-1

**Published:** 2026-07-13

**Authors:** S.K. Prajwal, Sushil K. Sharma, Narayan Sahu, Lalit Laxman Kharbikar, Sanjay K. Jain, Lata Jain, Aditi Kundu, Anil Dixit

**Affiliations:** 1 ICAR-National Institute of Biotic Stress Management, Raipur, Chhattisgarh 493225 India; 2 ICAR-Indian Agricultural Research Institute, Pusa, New Delhi, 110012 India

**Keywords:** *Bacillus cabrialesii*, Cyclic lipopeptides, *Fusarium oxysporum* f. sp. *ciceris*, Gum acacia, Defence-related biomolecules/enzymes, vermicompost, Chickpea

## Abstract

**Background:**

*Bacillus cabrialesii*, a plant growth-promoting (PGP) and biocontrol bacterium, was first reported in Mexico in 2018. This study evaluates the PGP and antagonistic potential of two *B. cabrialesii* strains, IS-10 and BATS-13, isolated from the chickpea rhizospheric soils of Chhattisgarh, India. It also assesses the impact of strain IS-10 on the growth, yield, and disease resistance of chickpea cultivars under *Fusarium* wilt stress.

**Methods:**

PGP traits and antifungal activity of the isolates were characterized in vitro, including biosurfactant production. The biosurfactant nature was analyzed using thin-layer chromatography (TLC) and ultra-performance liquid chromatography tandem mass spectrometry (UPLC-MS/MS). Strain IS-10 was used for seed bacterization in sterile soil amended with 0.2% vermicompost and 0.02% gum acacia, in the presence or absence of *Fusarium oxysporum* f. sp. *ciceris* (FOC), to evaluate its effects on plant growth and disease suppression in two chickpea cultivars, Vaibhav and JG-62.

**Results:**

Both strains demonstrated multiple PGP attributes and inhibited FOC growth in vitro. TLC revealed the presence of lipids and peptides in the crude biosurfactant, and further UPLC-MS/MS analyses confirmed the presence of surfactin and fengycin. In the pot trials, strain IS-10 treatment significantly improved plant performance. In pathogen-free conditions, strain IS-10 increased straw yield by 39.17% (Vaibhav) and 39.47% (JG-62), and grain yield by 46.8% (Vaibhav) and 50.32% (JG-62), respectively, over untreated controls. Under FOC challenge conditions, strain IS-10 reduced disease incidence by 20.80% (Vaibhav) and 26.85% (JG-62), while enhancing straw yield by 53.92% and 36.6%, and grain yield by 57.14% and 50.81%, respectively, compared to pathogen controls.

**Conclusions:**

*B. cabrialesii* IS-10 possesses significant PGP and biocontrol traits, including production of antifungal lipopeptides. Its application enhances chickpea growth and yield and mitigates the effects of Fusarium wilt, highlighting its potential as a bioinoculant for sustainable chickpea cultivation under both healthy and pathogen-infested conditions in the state of Chhattisgarh, India.

**Supplementary Information:**

The online version contains supplementary material available at https://doi.org/10.1186/s12866-026-05357-1.

## Introduction

Global food security is increasingly threatened by rapid population growth and the adverse effects of climate change on agriculture, which remains essential for sustaining human livelihoods and nutritional demands. Sustainable agricultural practices, including the incorporation of organic matter, use of cover crops, mulching, and conservation tillage or no-till systems, contribute significantly to improved soil health and enhanced carbon sequestration, thereby helping to mitigate climate change impacts. Pulses serve as highly nutritious food and feed sources and play an important role in reducing greenhouse gas emissions compared with many other cropping systems. Leguminous crops contribute to soil carbon sequestration, with carbon accumulation rates reported to begin at approximately 7.21 g C kg⁻¹ dry matter, resulting in 23.6 g C kg⁻¹ soil in maize–soybean intercropping systems compared with 21.8 g C kg⁻¹ soil in conventional maize monoculture systems over time. Furthermore, pulses reduce dependence on synthetic nitrogen fertilizers through biological nitrogen fixation, thereby lowering fossil fuel inputs and reducing carbon dioxide emissions by approximately 277 kg CO₂ ha⁻¹ [1].

Chickpea (*Cicer arietinum* L.) is cultivated globally, including India, due to its high nutritional value and can enhance soil fertility. It is rich in fiber, complex carbohydrates, calcium, phosphorus, iron, magnesium, vitamins and folate [2]. However, chickpeas encounter significant challenges from both biotic and abiotic stresses that adversely affect their yield. Major insect pests affecting chickpea cultivation include pod borer, black cutworm, semi-looper, aphids, leaf miners, and bruchids. In addition to insect pests, several phytopathogens pose serious constraints to chickpea production by causing economically important diseases. The major diseases of chickpea include Fusarium wilt, dry root rot, collar rot, Ascochyta blight, and Botrytis grey mold [3]. Among these, Fusarium wilt caused by *Fusarium oxysporum* f. sp. *ciceris* (FOC), either alone or in association with *Sclerotium rolfsii*, is particularly destructive and may result in severe yield losses, occasionally leading to complete crop failure under favorable disease conditions. Symptoms of Fusarium wilt can manifest at any growth stage of the chickpea plant, often resulting in either patchy or widespread plant death in fields [4]. The disease is more prevalent in drier growing regions compared to colder climates. This pathogen can be both soil- and seed-borne, making control measures challenging since fungicides are often ineffective against it. Breeding efforts have been made to develop resistant chickpea varieties that significantly reduce the impact of Fusarium wilt. Nevertheless, there is a risk of resistance breakdown at any time [5].

Management strategies for controlling Fusarium wilt should prioritize the reduction of pathogen inoculum levels. Several antagonistic microbes, such as *Penicillium*, *Trichoderma*, *Bacillus*, *Aspergillus*, *Streptomyces*, and *Pseudomonas*, have been identified as potential biocontrol agents [6, 7]. Among these, *Bacillus* species are particularly effective due to their ability to colonize plant roots and their diverse mechanisms of action [8]. Specific strains, such as *Bacillus fortis*, *B. thuringiensis*, and *B. subtilis*, have demonstrated promise in managing diseases across various crops, including chickpea [9, 10].

Recently, the discovery of *B. cabrialesii* TE3^T^ in the wheat rhizosphere of Mexico has demonstrated its potential for promoting plant growth and controlling diseases [11]. In India, various strains of *B. cabrialesii* have been isolated from different environments, including salt pans and wheat fields, suggesting their usefulness in biocontrol applications against soil-borne pathogens affecting chickpeas [12, 13]. Notably, in the state of Chhattisgarh, strains of *B. cabrialesii* have been reported for the first time in the rhizosphere soil of both wilted and healthy chickpea plants. Chickpeas are cultivated during the rabi season, following rice cultivation, and the residue of rice crop provides a favorable environment for various plant pathogenic soil-borne pathogens, particularly in Chhattisgarh, such as *Sclerotium*, *Macrophomina* and *Fusarium*. To effectively manage Fusarium wilt in chickpeas, an integrated strategy is necessary, combining resistant varieties with biocontrol agents such as the native *B. cabrialesii* strain. The present investigation aimed to evaluate the plant growth-promoting and biocontrol potential of two *Bacillus cabrialesii* strains, IS-10 and BATS-13, isolated from the rhizosphere soils of chickpea cultivated in the Chhattisgarh state of India. Furthermore, the study assessed the effect of strain IS-10 on the growth, disease protection, and yield performance of two chickpea cultivars under both challenged and non-challenged conditions with *Fusarium oxysporum* f. sp. *ciceris* (FOC).

## Materials and methods

### Isolation of *Bacillus cabrialesii* strains and fungal pathogens

*Bacillus cabrialesii* strains IS-10 (NCBI Accession No. PQ795767; Culture Deposit Accession No. NAIMCC-B-04214) and BATS-13 (NCBI Accession No. PQ795772; Culture Deposit Accession No. NAIMCC-B-04212) were isolated from the rhizosphere soil of chickpea plants in the Raipur (Latitude: 21°23.76’N; Longitude: 81°70.55’E) and Bemetra (Latitude: 21°76.54’N; Longitude: 81°47.76’E) districts of Chhattisgarh, India (Supplementary Figure 1). The soil-borne pathogen, FOC was collected from the Microbiology Laboratory of ICAR-NIBSM, Raipur, India.

### Assessment of plant growth-promoting abilities of *B. cabrialesii*

The *B. cabrialesii* strains were evaluated for their plant growth-promoting characteristics on a qualitative basis. Siderophore production was assessed using the Chrome Azurol S assay [14]. In brief, 10 µL of culture from each strain grown in the logarithmic phase was spot-inoculated on siderophore plates, followed by incubation at 28 ± 1 °C for 3 to 4 days. The presence of siderophores was indicated by the formation of a yellow or orange halo around the colonies. Phosphate solubilization potential was evaluated according to the methods described by Penmetsa et al. [2]. To conduct this test, bacterial cultures grown for 48 h were spotted with sterile toothpicks directly in the center of modified Pikovskaya agar plates, followed by incubation at 28 ± 1 °C for 5 to 7 days. Formation of a clear halo around the colonies indicated phosphate solubilization. The potential of *B. cabrialesii* strains to solubilize zinc was tested using a tris-minimal agar medium supplemented with filter-sterilized D-glucose as the carbon source and various insoluble zinc compounds, following the method described by Gurjar et al. [15]. The degree of zinc solubilization was evaluated by measuring the diameter of the halo zone around the colonies. Indole-3-acetic acid (IAA) production was assessed using the method outlined by Ahmad et al. [16]. Specifically, two strains were grown in Luria-Bertani (LB) broth, either with or without 200 µg/ml of L-tryptophan, at 28 ± 1 °C for 4 days while shaking at 120 rpm. Afterwards, the cultures were centrifuged, and 2 ml of the supernatant was mixed with orthophosphoric acid and Salkowski’s reagent. This mixture was incubated for one hour in the dark, followed by measuring the IAA concentration at 530 nm. To screen for ammonium production by the bacterial strains, 10 ml of sterile peptone water was inoculated with bacterial cultures grown for 48 h. The test tubes were then incubated at 28 ± 1 °C for 72 h on a rotary shaker at 120 rpm. After incubation, 0.5 ml of Nessler’s reagent was added. A color change from yellow to dark brown indicated the presence of ammonium, as noted by Alkahtani and co-workers [17].

### Evaluation of the biocontrol potential of *B. cabrialesii* strains

#### Confrontation test against FOC

To evaluate the ability of *B. cabrialesii* strains to inhibit *Fusarium oxysporum* f. sp. *ciceris* (FOC), the pathogen was cultured on potato dextrose agar (PDA) at 28 ± 1°C for a dual culture confrontation test. *B. cabrialesii*, grown overnight on nutrient agar (NA), was spot-inoculated at equal distances from the edge of a Petri dish containing a 1:1 mixture of PDA and NA agar. The dishes were then incubated for 48 h at 28 ± 1°C. After this incubation, a 5 mm disc of the fungal pathogen was placed in the center of the dish to allow for further growth. The percentage growth inhibition was calculated using the following formula: $\mathrm N\;=\;(\mathrm A\;-\;\mathrm B)/\mathrm A\;\mathrm x\;100$. In this formula, ‘N’ represents per cent (%) growth inhibition, ‘A’ is the colony diameter of the uninhibited FOC, and ‘B’ is the colony diameter of the treated FOC.

### Pathogen growth by the bacterial volatiles production

A five-millimetre (5 mm) disc culture of FOC was placed on one half of a divided Petri dish containing potato dextrose agar (PDA). Simultaneously, a 10 µL bacterial suspension (1.5 × 10^8^ CFU/mL) was streaked onto the other half of the dish, which contained nutrient agar (NA). Both the treated plates and control dishes, which did not contain bacteria, were then incubated at 30 °C for 7 days. After the incubation period, fungal growth was measured. Each strain was tested in triplicate, following the modified methodology described by Khalifa et al. [18]. To calculate the per cent (%) growth enhancement, the following formula was used: $\mathrm N=(\mathrm B-\mathrm A)/\mathrm A\times100$, where ‘N’ represents the growth enhancement (%); where ‘A’ is the colony diameter of the control FOC and ‘B’ is the colony diameter of the treated FOC.

### Hydrogen cyanide production (HCN)

Hydrogen cyanide production was recorded after 72 h incubation of bacterial strains on NA at 28 ± 1 °C. The production of HCN involved filter paper soaked in alkaline picrate, placed inside a Petri dish. The subsequent color change from yellow to reddish-brown indicated the degree of cyanogenic potential [19].

### Hydrolytic enzyme production

The protease production assay involved inoculating overnight-grown strains of *B. cabrialesii* onto skim milk agar, as outlined by Lork [20]. The cultures were incubated in the dark at a temperature of 28 ± 1 °C for 24 to 48 h to evaluate protease production. This was indicated by the formation of a clear halo around the colonies. The potential for protease production by the *B. cabrialesii* strains was assessed by measuring the size of the halo zone surrounding the bacterial colonies. Additionally, an amylase production assay was conducted by inoculating the overnight-grown *B. cabrialesii* strains onto starch agar, following the method mentioned [21]. These cultures were incubated at 37 ± 1 °C for 48 h. Subsequently, 2–3 drops of iodine solution were added directly, and the development of a halo zone after 10–15 min indicated amylase production. For the cellulase production assay, overnight-grown strains of *Bacillus cabrialesii* were inoculated onto M9 minimal agar medium supplemented with D-glucose and 0.2% carboxymethyl cellulose (CMC). The cultures were then incubated at 28 °C for five days. After incubation, the plates were flooded with a 0.1% Congo red solution for 30 min at room temperature, followed by washing with 1 M NaCl. The formation of a clear halo around the colonies indicated a positive response [22]. The lipase production assay involved inoculating the overnight-grown *B. cabrialesii* strains onto M9 salt agar medium supplemented with D-glucose and 1% Tween 20. After 6 days of incubation at 30 °C, the appearance of crystals along with a halo indicated the production of lipase [23].

Finally, to assay for chitinase production, the overnight-grown *B. cabrialesii* strains were inoculated onto M9 minimal agar medium supplemented with D-glucose and 0.4% colloidal chitin [24]. A halo zone around the colonies after incubation for 14 days at 30 ± 1 °C was considered indicative of chitinase-producing strains.

### Screening for biosurfactant (lipopeptide) production by the *B. cabrialesii* strain

Four screening methods were carried out to determine whether the selected strains could produce biosurfactants. The methods used included: (1) Oil Spreading Method, (2) Emulsification Index, (3) Drop Collapse Method, and (4) Parafilm M Test. For the oil spread test, a Petri dish was filled with 20 ml of distilled water, followed by placing a layer of 20 ml of crude oil on the water’s surface. After this, 10 ml of cell-free culture broth was added. Formation of a clear zone on the oil indicated the positive nature of the biosurfactant. In this experiment, water was used as the negative control, while Triton served as the positive control. According to Sarubbo et al. [25], the emulsification activity of the cell-free extract was assessed by filling a test tube with 2.0 mL of cell-free extract and 2.0 mL of kerosene. The mixture was then vortexed vigorously for 2 min to ensure proper homogenization. After 24 h, the emulsification activity was calculated using the formula provided by Lai et al. [26]: $$\begin{aligned} &\mathrm E24\;(\%)=(\mathrm{Total\;height\;of\;the\;emulsified\;layer}(\mathrm{mm})/ \\& \mathrm{Total\;height\;of\;the\;liquid\;layer}(\mathrm{mm}))\times100 \end{aligned}$$.

Additionally, the drop collapse assay was performed using the method described by Bodour et al. [27]. Crude oil was placed in the wells of a 96-well microplate and allowed to settle for 24 h at room temperature. Following this, 5 mL of cell-free extract was added to the oil-coated wells, and the size of the oil drops was measured under a microscope after one min. Biosurfactant activity was defined as drops with diameters greater than 1 mm, with 1 mg/mL Triton X-100 used as the control. If the oil drop collapsed after the addition of the cell-free extract, it is considered positive for the presence of biosurfactant. The Parafilm M test was carried out by taking 25 µL (25 µL) of cell-free extract on the hydrophobic surface of a Parafilm M strip. The presence of surface-active compounds in the extract after one min was indicated by a flattened droplet shape, while a semi-spherical shape suggested the absence of a biosurfactant [28].

### Detection of the biosurfactant nature of cell-free extract by TLC

Thin-layer chromatography (TLC) was conducted to preliminarily characterize the biosurfactants. A small amount of crude biosurfactant concentrate, rich in surfactin and fengycin, was dissolved in methanol and loaded onto the TLC plate. The mobile phase was a mixture of chloroform, methanol, water, and glacial acetic acid in the ratio of 65:15:2:1 (v/v/v/v). To visualize the spots formed on the silica gel plates, various color-developing reagents were sprayed onto them. Iodine crystals were used to identify the lipid portion of the biosurfactant. For the visualization of protein and carbohydrate spots, ninhydrin reagent (0.25% ninhydrin in acetone) and Molisch reagent (5% 1-naphthol in alcohol) were employed, respectively. The silica gel plates were heated in a hot air oven at 110 °C for 10 min to visualize the spots [29].

### UPLC-ESI-MS/MS analysis

The composition analysis of biosurfactants was conducted using Ultra-Performance Liquid Chromatography (UPLC) combined with a photodiode array (PDA) and a mass spectrometer. The stationary phase used was a C18 column (2.1 mm × 100 mm, 1.8 μm; Waters, Milford, MA, USA) for the separation of compounds, whereas the mobile phase consisted of (A) acetonitrile (ACN) with 0.1% formic acid (FA) and (B) water with 0.1% FA. An injection volume of 5 µL was utilized for analysis purposes. The gradient program commenced with 60% solution A for 3 min, followed by a transition from 60% to 10% A over 3 to 18 min, and concluded with 60% A from 18 to 20 min. Two standards, surfactin and fengycin, were prepared separately, and their corresponding regions were used to create a calibration curve.

Mass data were acquired using a Waters ACQUITY UPLC^®^ system coupled with a mass spectrometer equipped with an electrospray ionization (ESI) source and a hybrid Quadrupole-Time of Flight (QToF-MS) (Waters Corporation, Manchester, UK). The UPLC-QToF-MS/MS system was controlled using the advanced Mass Lynx 4.3 software. Data acquisition was conducted using the MSE function in continuum mode, covering a mass-to-charge (m/z) range of 50 to 2000. The MSE mode enables the simultaneous collection of full scan MS data at low energy (6 V) and MS/MS data at high energy, with a ramp ranging from 20 to 60 V.

The following source parameters were set: desolvation gas flow at 500 L/h, cone gas flow at 40 L/h, capillary voltage at 3.5 kV, sampling cone voltage at 30 V, extraction cone voltage at 6 V, source temperature at 15 °C, and desolvation temperature at 25 °C. Sodium formate (0.5 mM) was used to calibrate the mass spectrometer. A flow rate of 20 µL/min with a concentration of 1 µg/mL was employed for the lock spray every 10 s, using leucine enkephalin (m/z 556.2771 in positive polarity and m/z 554.2670 in negative polarity) as the reference mass for mass correction.

### Effects of *B. cabrialesii* IS-10 on plant growth promotion and control of FOC-induced wilt disease control

#### Pot culture

A pot experiment was conducted using a randomized block design with two chickpea cultivars, Vaibhav and JG-62, and included six treatments along with four replications. The treatments were as follows: (1) T1 (Control): Sterilized soil + Sterilized vermicompost (5 tons/ha or 0.2%), (2) T2: Sterilized soil + Sterilized vermicompost + *B. cabrialesii* IS-10 (1 mL of 10⁸ CFU/mL), T3: Sterilized soil + Sterilized vermicompost + Sterilized gum acacia (1.5% aqueous solution, sterilized or 0.02% or 5.8 quintals/ha) + *B. cabrialesii* IS-10 (1 mL of 10⁸ CFU/mL), T4: Sterilized soil + Sterilized vermicompost + FOC, T5: Sterilized soil + Sterilized vermicompost + FOC + *B. cabrialesii* IS-10 (1 mL of 10⁸ CFU/mL), T6: Sterilized soil + Sterilized vermicompost + FOC + Gum acacia (1.5% aqueous solution, sterilized or 0.02% or 5.8 q/ha) + *B. cabrialesii* IS-10 (1 mL of 10⁸ CFU/mL). The experiment contained 48 pots, each containing 15 kg of sterilized soil. The soil was collected from the ICAR-National Institute of Biotic Stress Management Research Farm in Raipur and was subjected to a 2 mm sieve before being sterilized through Tyndallization in an autoclave on two separate occasions, one day apart. Each pot received 33.48 g of sterilized dried vermicompost. The pathogen inoculum of *Fusarium oxysporum* f. sp. *ciceris* was mass multiplied for 20 days on sterilized barley grains and incorporated into the potting substrate at 2% (w/w) after grinding in a grinder for 5 min, corresponding to approximately 300 g inoculum per potting mixture, resulting in an inoculum density of 1.3–1.6 × 10⁵ CFU spores and propagules g⁻¹ soil. Inoculum of FOC was then added to treatments T4, T5, and T6 and left to stabilize for a week. The experimental soil had the following attributes: Sand-24.33%, silt-22.71%, clay-51.87%, pH-7.4, EC-0.21 dS/m, available nitrogen-153.21 µg/g, available phosphorus-8.80 µg/g, potassium-268 µg/g, sulfur-8.56, Zn-0.51 µg/g, Fe-2.10 µg/g, Cu- 0.41 µg/g and Mn-1.28 µg/g. The vermicompost used contained 1.8% nitrogen, phosphorus, and 1.2% potassium, along with various micronutrients and organic carbon levels ranging from 12.54%.

### Preparation of fungal inoculum

Fifty grams (50 g) of barley seeds were ground into small particles and placed in a 250 ml conical flask. The ground seeds were then mixed with enough water and 5 gm jaggery to achieve proper moisture content and subjected to sterilize After autoclaving, 5 to 6 plugs (5 mm) of FOC were inoculated into the conical flask, and then the flask was incubated in a BOD incubator for 20 days to allow complete colonization of the substrate by the pathogen.

### Preparation of bacterial inoculum

The effects of *B. cabrialesii* IS-10 were evaluated on plant growth, disease incidence, rhizosphere microbial properties, and the growth and yield of plants. To prepare liquid cultures, a freshly grown colony of the bacteria was transferred to a nutrient broth and shaken at 150 rpm while being incubated at 28 ± 2 °C for 48 h. After incubation, the fully-grown bacterial culture was centrifuged for 15 min at 6000 rpm. The resulting pellets were then suspended in a sterile saline solution (0.85% sodium chloride, pH 5.5) to achieve a concentration of 10^8^ CFU/mL.

### Preparation of a plant gum solution

Natural gum acacia (*Acacia nilotica*) was purchased from the local Gol market of Raipur. Gum acacia consists primarily of L-arabinose, L-rhamnose, d-glucuronic acid, and 1,3-linked β-D-galactopyranosyl, along with other minor compounds. To prepare an aqueous solution, 3.0 g of gum acacia was added to 200 milliliters of double-distilled water. This solution was then sterilized in an autoclave. A total of 100 milliliters of the sterilized solution was mixed into each respective pot.

### Biometrical observations

During the seedling stage of growth, we recorded key indicators such as germination percentage, dry shoot weight, and dry root weight. These important metrics were assessed to evaluate the plants’ vitality and to gain valuable insights into their overall health.

### Evaluation of disease severity and incidence

Each plant received a severity score based on the observed symptoms, as stated by Khalifa et al. [18]. The scoring scale (No symptoms: 00; Yellowing or wilting of 1/3 of the plant: 01; Yellowing or wilting of 2/3 of the plant: 02; 3: Yellowing or wilting of the entire plant: 03; The plant is dead: 04) has been recorded. The overall disease severity (DS) was calculated using the following formula: $\mathrm{DS}\;(\%)=\lbrack(\mathrm{\sum\;v}\times\mathrm n)/(\mathrm N\times\mathrm V)\rbrack\times100$ . In this formula, ‘n’ represents the number of plants assigned to each severity score, ‘v’ is the severity score, ‘V’ is the maximum severity score, and ‘N’ is the total number of plants evaluated. Disease incidence was calculated according to the formula provided by Khalifa et al. [18]: $\mathrm{Disease}\;\mathrm{incidence}=\mathrm{Total}\;\mathrm{number}\;\mathrm{of}\;\mathrm{diseased}\;\mathrm{plants}/\mathrm{Total}\;\mathrm{number}\;\mathrm{of}\;\mathrm{plants}$.

### Estimation of chlorophyll content in leaves

The total chlorophyll content of fresh leaves at the pre-flowering and flowering stages was measured using the dimethyl sulfoxide (DMSO) method, as outlined by Hiscox and Israelstam [30]. In summary, 50 mg of thoroughly cleaned and chopped fresh leaves were placed into a glass test tube containing 10 mL of DMSO. The mixture was then incubated at 65 °C for 3 h in a hot air oven. After incubation, the volume was adjusted to 10 mL by adding more DMSO. The contents were allowed to settle, and the absorbance of the supernatant was measured at wavelengths of 645 nm and 663 nm.

### Dehydrogenase activity (DHA) of soil at pre-flowering and flowering stages

DHA was determined using a colorimetric method that reduces 2,3,5-triphenyl tetrazolium chloride (TTC) to triphenyl formazan (TPF) [31]. In this procedure, 1 g of moist soil was placed in a screw-cap glass tube along with 0.2 mL of 3% TTC and 0.5 mL of 1% glucose. The contents of the tube were mixed thoroughly before being incubated at 35 °C in a hot air oven for 24 h. After incubation, 10 mL of methanol was added to the tube, and the mixture was stirred before being refrigerated for 3 h. Finally, the red color of the TPF was measured at 485 nm using a spectrophotometer, and the enzyme activity was expressed in pico Katal per gram of soil (pKat/ g) on a dry weight basis.

### Soluble protein estimation

Samples of leaves and stems were placed in liquid nitrogen and stored in a freezer at -80 °C. A 100 mg sample was homogenized using an ice-cold 0.1 M sodium phosphate buffer (pH 7.0) in a chilled mortar and pestle. The homogenate was then centrifuged in a 2 mL Eppendorf tube at 15,000 rpm for 15 min at 4 °C. The collected supernatant was used to estimate the total protein level and enzyme activities. The total soluble protein in the samples was estimated using the dye-binding method described by Bradford [32].

### Superoxide dismutase (SOD) enzyme assay

The activity of SOD in the leaves and stems of chickpea cultivars was determined using nitro-blue tetrazolium (NBT) as a reagent [33]. SOD activity was measured by monitoring the gradual decrease in optical density (OD) of formazan, which is produced when the superoxide radical reacts with the NBT dye in the presence of the enzyme. To initiate the reaction, 0.1 mL of a 2 µM riboflavin solution was added to test tubes, which were then placed beneath two 15 W fluorescent lamps for 15 min to promote the reaction. A control mixture lacking the enzyme was included to provide a baseline for maximal color intensity. Once the reaction time was completed, the light was turned off to halt the reaction, and the tubes were then kept in the dark. A non-irradiated complete reaction mixture was used as a blank for comparison. The absorbance was recorded at 560 nm, and enzyme activity was expressed as the amount of enzyme needed to reduce the absorbance to 50% of the level observed in the control. This unit was then converted to express enzyme activity in micromoles per minute per milligram of protein (µmol/min/mg protein).

### Catalase activity assay

The catalase activity was performed by mixing phosphate buffer (100 µM, pH 7.0) and hydrogen peroxide (H_2_O_2_, 100 µM, pH 7.0) with the addition of 1 ml of enzyme extract employing the method of Abi [34]. The mixture was allowed to react in the dark for 1 min, during which the enzymatic decomposition of hydrogen peroxide (H₂O₂) released oxygen (O₂). The amount of O₂ produced was quantified by measuring the absorbance at 240 nm, using the extinction coefficient of H₂O₂, which is 0.036 nM cm⁻¹. The enzyme activity was expressed as the amount of H₂O₂ decomposed per minute per milligram of protein.

### Peroxidase activity assay

Peroxidase activity was measured by mixing 1.5 mL of pyrogallol (0.05 mol), 0.5 mL of hydrogen peroxide (1% v/v), and 0.05 mL of the enzyme extract, following the method described by Nakano et al. [35]. The absorbance (optical density) was recorded at 420 nm at 30-s intervals. Enzyme activity was expressed as micromoles of hydrogen peroxide reduced per minute per milligram of protein (µmol H₂O₂ reduced/min/mg protein).

### Phenylalanine ammonia-lyase (PAL) activity assay

PAL activity was assessed using an enzyme-linked assay that converts phenylalanine to trans-cinnamic acid, which was measured spectrophotometrically at 290 nm, as described by Campos et al., [36]. In brief, 0.2 g of root tissue was macerated in 2 ml of 50 mM borate buffer (pH 8.5) containing 0.4 g of insoluble polyvinyl polypyrrolidone and 5 mM of 2-mercaptoethanol. The mixture was then centrifuged at 12,000 rpm at 4 °C for 20 min, and the resulting supernatant was used as the enzyme source. To measure PAL activity, a 0.1 mL aliquot of the supernatant was mixed with 0.9 mL of distilled water and 1 mL of 100 mM L-phenylalanine and incubated at 40 °C for 30 min. Finally, the reaction was stopped by adding 1 mL of 4% trichloroacetic acid (TCA). A blank was prepared by adding TCA to a separate test tube before the reaction began (at zero minutes). After the reaction was terminated, the mixture was centrifuged at 10,000 g for 5 min, and the absorbance was recorded at 290 nm. PAL activity was expressed as nmol trans-cinnamic acid produced per mg of protein per minute.

### Biometrical observations at the harvesting stage

The crops were harvested 84 days after sowing, at which time we measured plant height, root weight, root length, pod numbers, straw yield, and grain yield.

### Statistical analyses

To evaluate the significance of the plant growth-promoting and biocontrol properties of *Bacillus cabrialesii* IS-10 and *B. cabrialesii* BATS-13 under in vitro conditions, as well as the effects of *B. cabrialesii* IS-10 on plant growth, wilt disease suppression, soil dehydrogenase activity, defense responses, and yield-related traits in chickpea cultivars Vaibhav and JG-62, pot experiments were conducted with four biological replicates. Data were analyzed using one-way analysis of variance (ANOVA) through OPSTAT software developed by O.P. Sheoran, CCS Haryana Agricultural University (CCS HAU), Hisar, India. Mean comparisons were performed using the least significant difference (LSD) test at a 95% significance level (*p* ≤ 0.05). Results are presented as mean ± standard deviation (SD). Graphs were generated using Microsoft Excel (Windows 10).

## Results

### Cultural characteristics of *Bacillus cabrialesii* strains

Two strains, namely IS-10 and BATS-13 of *B. cabrialesii*, from Raipur and Bemetara districts, respectively, were examined for their colony morphology. Both strains were medium-sized, irregularly shaped, and had a rough surface texture. The strain IS-10 exhibited a wavy margin and a raised elevation, whereas the strain BATS-13 had a lobate margin with a similar elevation. Both strains were found to be off-white, Gram-positive, and produced endospores (Supplementary Table 1). In terms of biochemical characteristics, both strains IS-10 and BATS-13 tested positive for citrate utilization, catalase, oxidase, and nitrate reduction. However, they tested negative for the indole test (Supplementary Table 2).

### PGP and biocontrol traits of *B. cabrialesii* strains

**Fig. 1:**
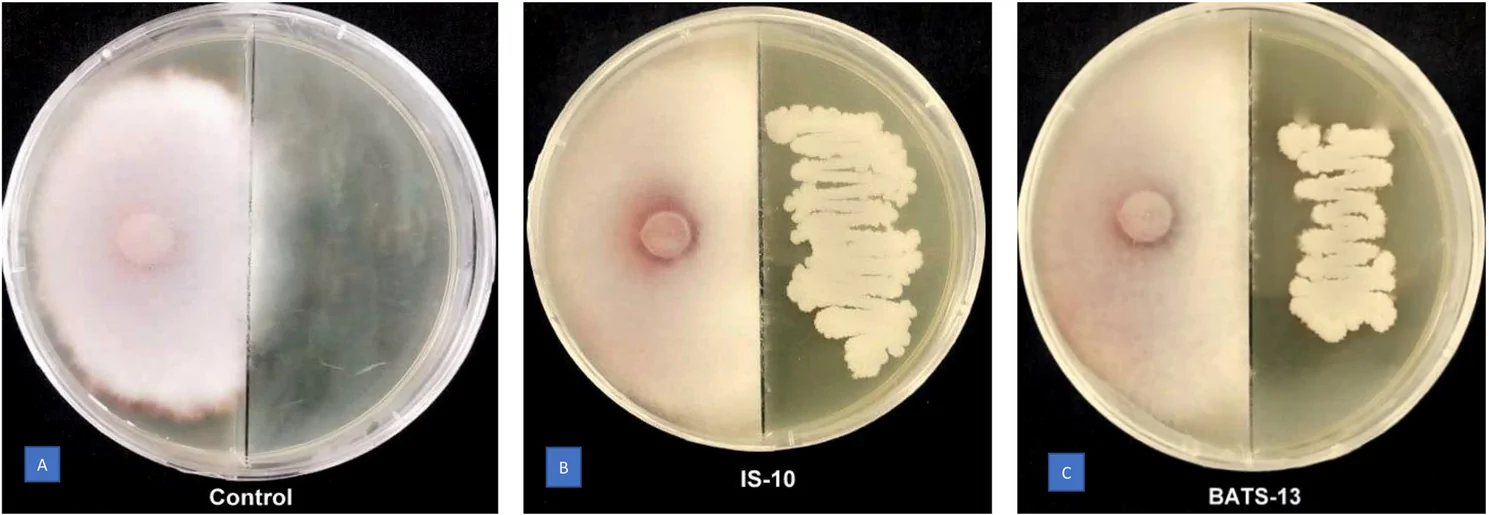
Enhancement of *F. oxysporum* f. sp. *ciceris* growth by volatiles of *B. cabrialesii* strains. **A**: Control (only *F. oxysporum* f. sp. *ciceris*), (**B**): Enhancement of *F. oxysporum* f. sp. *ciceris* by volatiles of *B. cabrialesii* IS-10, (**C**): Enhancement of *F. oxysporum* f. sp. *ciceris* by volatiles of *B. cabrialesii* BATS-13

Both strains produced IAA, siderophore, ammonium, protease, amylase, lipase, and cellulase (Table 1). They were found to solubilize tricalcium phosphate and zinc phosphate, but did not solubilize zinc oxide or zinc carbonate, though they did solubilize zinc phosphate. The results indicated that strain IS-10 was superior to strain BATS-13 with respect to traits. *B. cabrialesii* IS-10 produced higher levels of IAA (9.86 ± 0.65 µg/mL), siderophore (68 ± 0.95%), and solubilized more zinc phosphate (36.8 ± 0.40 µg/mL) compared to strain BATS-13. However, strain BATS-13 produced more ammonia (3.58 ± 0.24 µM/mL) than IS-10 (3.003 ± 0.27 µM/mL) (Supplementary Table 3; Supplementary Figure 2). The *B. cabrialesii* strains IS-10 and BATS-13 were evaluated for their antagonistic activity against *Fusarium oxysporum* f. sp. *ciceris* (FOC). Among the two strains, IS-10 exhibited comparatively higher antagonistic potential, showing 61.0% inhibition of FOC growth, whereas BATS-13 recorded 58.45% inhibition. Neither *B. cabrialesii* IS-10 nor BATS-13 exhibited chitinase activity or hydrogen cyanide (HCN) production, indicating the absence of these biocontrol traits. However, both strains produced volatile compounds that enhanced FOC growth under in vitro conditions. This observation remains intriguing and warrants further investigation, as most volatile organic compounds (VOCs) reported from *Bacillus* species are generally inhibitory toward phytopathogens (Table 1; Fig. 1).

**Table 1:** Plant growth promoting and biocontrol traits of *B. cabrialesii* strains

Traits		B. cabrialesii strains	
		IS-10	BATS-13
IAA production		+ (Pink colour)	+ (Pink colour)
Ammonia production		+ (Yellow colour)	+ (Yellow colour)
Phosphate solubilization (PSI)		+(3.4)	+(3.2)
Zinc solubilization (ZSI)	Zinc oxide	-	-
	Zinc phosphate	+ (4.6)	+ (4.0)
	Zinc carbonate	-	-
Siderophore production (SPI)		+ (2.41)	+ (2.14)
Protease production (PPI)		+ (2.13)	+ (2.82)
Amylase production (API)		+ (2.91)	+ (2.86)
Lipase production (LPI)		+ (2.14)	+ (2.04)
Cellulase production (CPI)		+ (2.21)	+ (2.11)
Antagonistic to *F. oxysporum* f. sp. *ciceris* (% inhibition)		61.00 ± 0.22	58.45 ± 0.17
Chitinase production		-	-
HCN production		-	-
Volatile production (Enhancement percentage of growth of *F. oxysporum* f. sp. *ciceris*)		6.34 ± 0.03	6.23 ± 0.02

### Characterization of the biosurfactant properties of cell-free extracts produced by *B. cabrialesii* strains

**Fig. 2:**
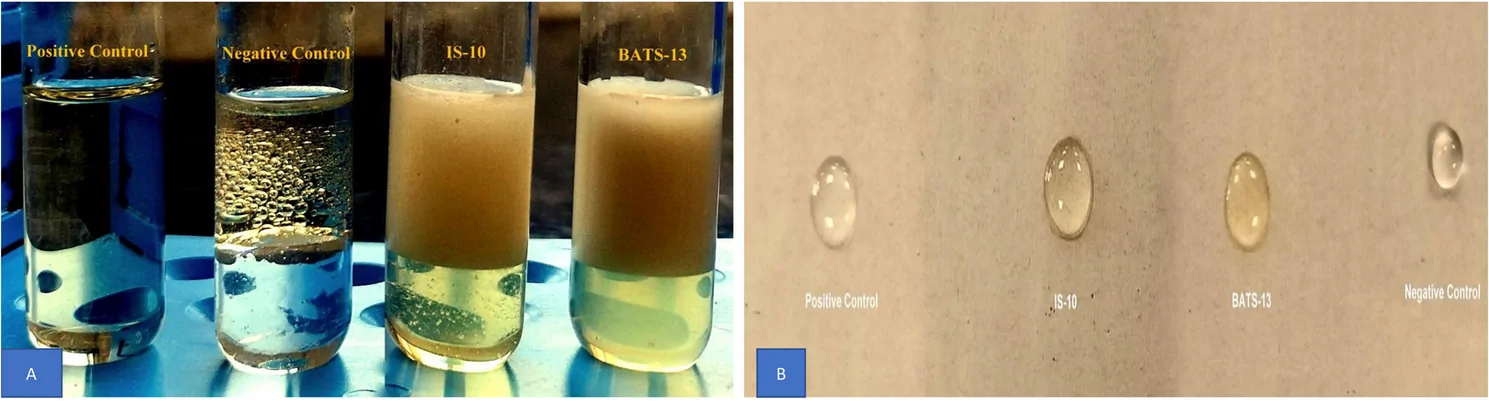
Biosurfactant production test of *B. cabrialesii* strains (**A**) Emulsifying activity of *B. cabrialesii* IS-10 & *B. cabrialesii* BATS-13, (**B**) Parafilm test of *B. cabrialesii* IS-10 & *B. cabrialesii* BATS-13

**Fig. 3:**
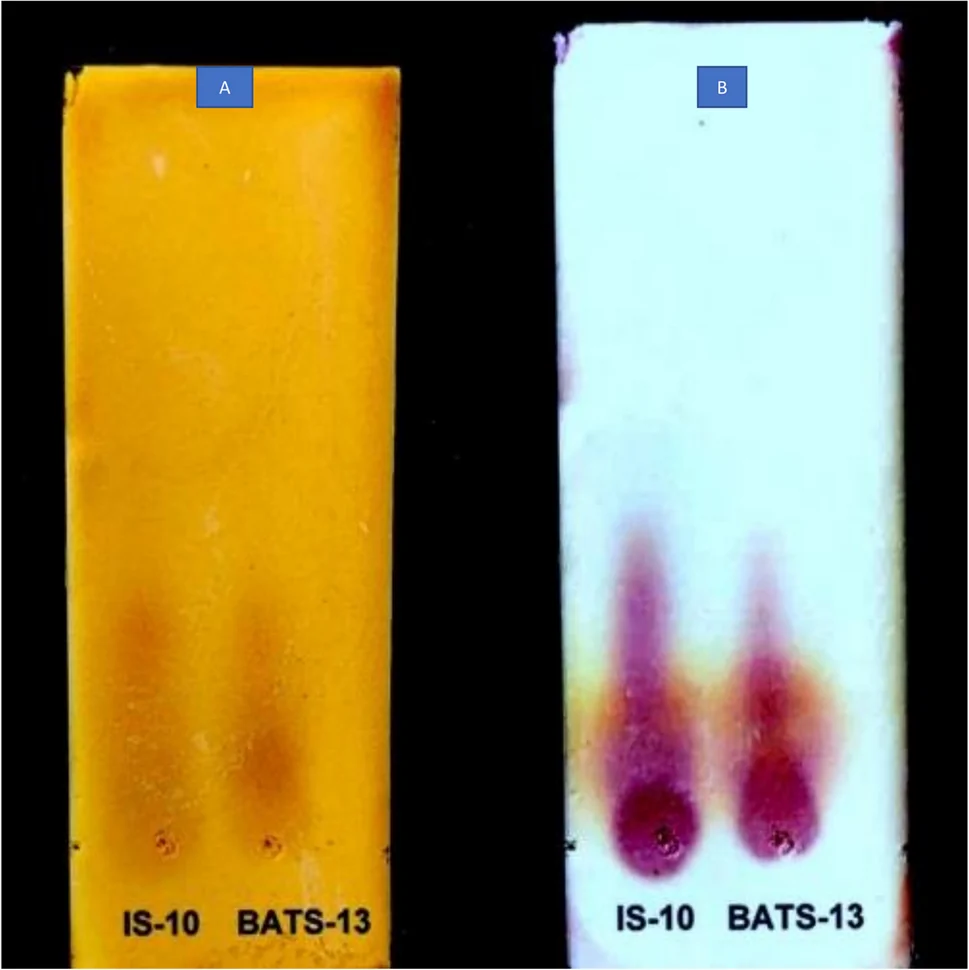
TLC plates indicating presence of Lipid (**A**) and Peptide (**B**) in biosurfactant of IS-10 & BATS-13

**Fig. 4:**
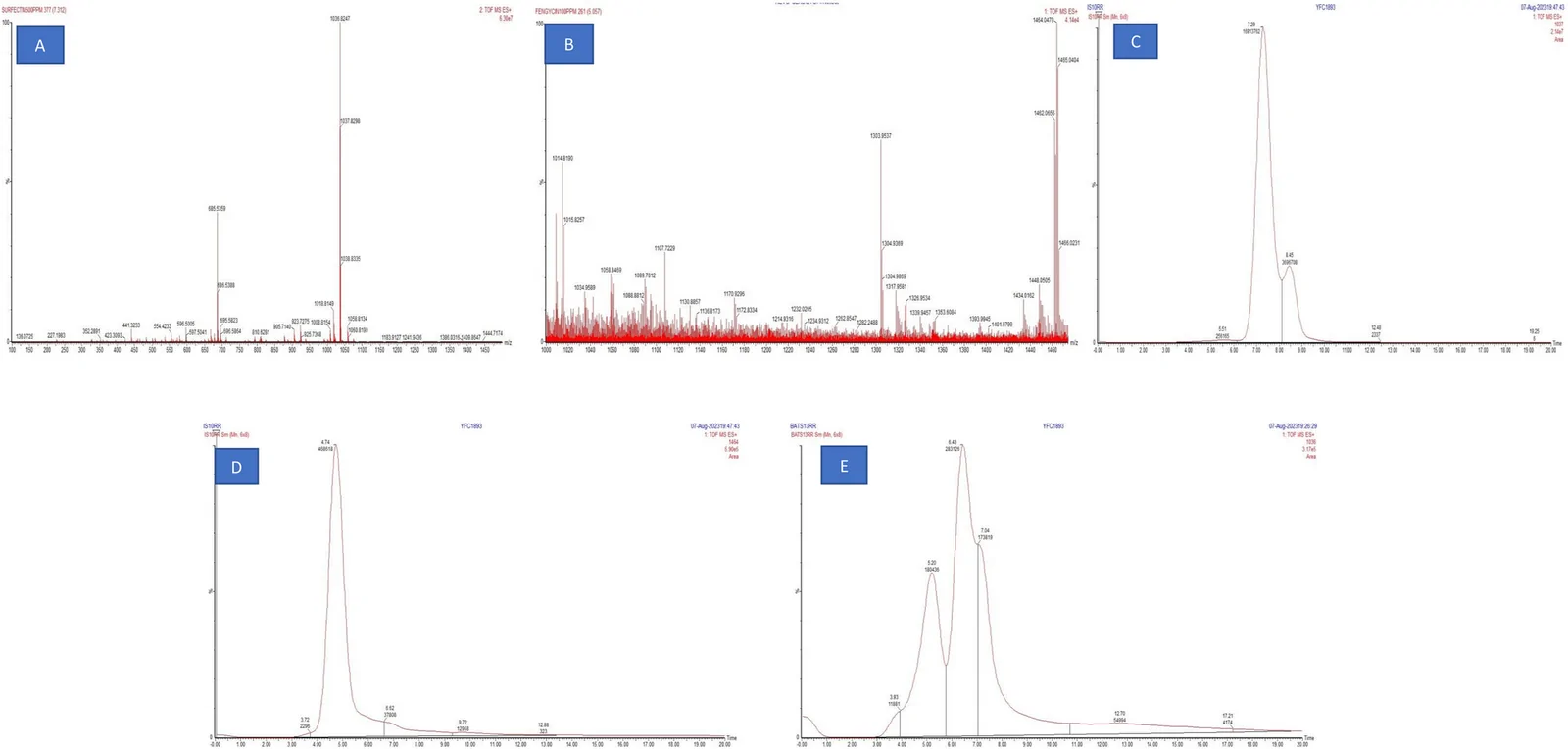
Lipopeptide detection by UPLC-MS (**A**): Standard curve of surfactin, (**B**): Standard curve of fengycin, (**C**): Presence of surfactin in *B. cabrialesii* IS-10 (**D**) Presence of fengycin in *B.cabrialesii* IS-10, (**E**) Presence of surfactin in *B.cabrialesii* BATS-13

Generally, cell-free extract showed positive results revealing biosurfactant nature of cell-free extract in several assays namely oil dispersion, emulsion stability, droplet collapse, and the parafilm test. Among tested strains, strain IS-10 demonstrated the highest activity (Table 2; Fig. 2). In the paper disc assay, the biosurfactant produced by *B. cabrialesii* was tested against FOC, showing a minimum inhibitory concentration (MIC) of 50 µg/mL for both strains. This indicates that the biosurfactants effectively function against the pathogen at lower concentrations (Table 3, Supplementary Figure 3). The crude biosurfactants produced by the strains IS-10 and BATS-13 were initially characterized using thin-layer chromatography (TLC). Overall, the results revealed the presence of lipids, indicated by a brown spot on exposure to iodine vapor, and peptides, visualized as a pink spot after ninhydrin exposure. This suggests that the biosurfactant has a lipoprotein/lipopeptide nature (Fig. 3). Following the initial characterization, the extracted biosurfactants were analyzed using Ultra Performance Liquid Chromatography-Electrospray Ionization-Mass Spectrometry (UPLC-ESI-MS/MS). The analysis confirmed the presence of surfactin and fengycin lipopeptides in both *B. cabrialesii* strains, IS-10 and BATS-13, as indicated by characteristic ion peaks detected at approximately m/z 1036.6 and m/z 1463.8, respectively, in the chromatogram (Fig. 4).

**Table 2:** Biosurfactant production of *B. cabrialesii* strains

Biosurfactant production tests	Strains	
	IS-10	BATS-13
Oil dispersion assay (cm)	2.83 ± 0.39	2.06 ± 0.21
Emulsification index (%)	62.26 ± 0.77	57.3 ± 0.38
Drop collapse assay	++	+++
Parafilm M test	+	+

**Table 3:** Paper disc assay of *B. cabrialesii* strains against *F. oxysporum* f. sp. *ciceris*

Concentration of crude biosurfactant (µg/mL)	Inhibition (%) of *F. oxysporum* f. sp. *ciceris*	
	IS-10	BATS-13
50	15.6 ± 0.35	11.34 ± 0.39
100	27.6 ± 0.67	22.4 ± 0.72
200	48.76 ± 0.64	36.8 ± 0.50
300	59.9 ± 0.69	52.9 ± 0.51

### Pot experiments

#### Agronomic attributes at the seedling stage

In the chickpea cultivar Vaibhav (RG 2918), the control treatment (T1) recorded 85.0% seed germination, a dry shoot weight of 159.7 mg plant⁻¹, and a dry root weight of 28.75 mg plant⁻¹ (Table 4). Inoculation with *B. cabrialesii* IS-10 in sterilized soil amended with vermicompost (T2) increased seed germination to 89.0% and significantly enhanced dry root weight to 36.75 mg plant⁻¹, representing a 27.8% increase over the control. The highest growth-promoting effect was observed when IS-10 was applied together with sterilized gum acacia (T3), resulting in 100% seed germination, a significant increase in dry shoot weight to 183.5 mg plant⁻¹ (14.9% over the control), and a dry root weight of 38.50 mg plant⁻¹ (33.9% over the control). Challenge inoculation with *Fusarium oxysporum* f. sp. *ciceris* (FOC) (T4) significantly reduced seed germination to 80.3% and decreased dry shoot weight to 148.75 mg plant⁻¹. However, the introduction of IS-10 in pathogen-infested soil improved seed germination to 87.5% (T5) and 91.6% (T6), respectively. The combined application of IS-10 and gum acacia in the presence of FOC (T6) resulted in the highest dry shoot weight (172.75 mg plant⁻¹) and dry root weight (40.25 mg plant⁻¹) among the pathogen-challenged treatments, indicating a substantial mitigation of Fusarium wilt-associated growth suppression.

**Table 4:** Effect of *Bacillus cabrialesii* IS-10 inoculation on seedling growth attributes (16 days after sowing) of chickpea cultivars Vaibhav (RG 2918) and JG-62 under non-challenged and *Fusarium oxysporum* f. sp. *ciceris*-challenged conditions

Treatments	Vaibhav (RG 2918)			JG-62		
	Seed germination (%)	Dry shoot weight (mg/plant)	Dry root weight (mg/plant)	Seed germination (%)	Dry shoot weight (mg/plant)	Dry root weight (mg/plant)
T 1	85 ± 1.83^bc^	159.7 ± 10.72^bc^	28.75 ± 7.44	95.83 ± 3.83a^b^	149.75 ± 11.00^a^	19.75 ± 1.83^b^
T 2	89 ± 1.63^bc^	167 ± 7.96^b^	36.75 ± 4.20^b^	95.83 ± 4.07^a^	114.25 ± 8.98^d^	23.50 ± 1.32^a^
T 3	100 ± 0.00^a^	183.5 ± 6.55^a^	38.50 ± 2.27^a^	95.83 ± 6.34^a^	136.25 ± 5.56^b^	18.25 ± 1.84^bc^
T 4	80.3 ± 0.75^c^	148.75 ± 8.04^c^	37.75 ± 1.99^a^	83.30 ± 5.29^c^	77.5 ± 5.20^e^	13.75 ± 2.56^d^
T 5	87.5 ± 0.98^bc^	157.75 ± 6.21^b^	36.25 ± 1.72^a^	87.50 ± 4.68^c^	81.25 ± 4.52^e^	14.00 ± 1.03^d^
T 6	91.6 ± 0.90^b^	172.75 ± 18.25^a^	40.25 ± 3.46^a^	87.50 ± 6.70^c^	126.66 ± 8.73^c^	16.50 ± 1.64^cd^
LSD (*p* ≤ 0.05)	8.18	15.67	6.02	7.88	8.46	2.64

Values are presented as mean ± SD (*n* = 3). Means within a column followed by different lowercase letters differ significantly according to Duncan’s Multiple Range Test (DMRT) at *p* ≤ 0.05; T1: Sterilized soil + sterilized vermicompost (0.2%; control); T2: Sterilized soil + sterilized vermicompost (0.2%) + *B. cabrialesii* IS-10 (1 mL of 10⁸ CFU mL⁻¹); T3: Sterilized soil + sterilized vermicompost (0.2%) + sterilized gum acacia (0.02%) + *B. cabrialesii* IS-10 (1 mL of 10⁸ CFU mL⁻¹); T4: Sterilized soil + sterilized vermicompost (0.2%) + *Fusarium oxysporum* f. sp. *ciceris* (FOC); T5: Sterilized soil + sterilized vermicompost (0.2%) + FOC + *B. cabrialesii* IS-10 (1 mL of 10⁸ CFU mL⁻¹); T6: Sterilized soil + sterilized vermicompost (0.2%) + FOC + sterilized gum acacia (0.02%) + *B. cabrialesii* IS-10 (1 mL of 10⁸ CFU mL⁻¹)

In the susceptible cultivar JG-62, the control treatment (T1) recorded 95.83% seed germination, 149.75 mg plant⁻¹ dry shoot weight, and 19.75 mg plant⁻¹ dry root weight. Application of IS-10 with vermicompost (T2) maintained germination at 95.83% and significantly increased dry root weight to 23.50 mg plant⁻¹ (19.0% over the control), although dry shoot weight declined to 114.25 mg plant⁻¹. The addition of gum acacia (T3) maintained high germination (95.83%) and improved dry shoot weight to 136.25 mg plant⁻¹, while dry root weight remained comparable to the control. Pathogen inoculation (T4) markedly reduced germination (83.30%), dry shoot weight (77.50 mg plant⁻¹), and dry root weight (13.75 mg plant⁻¹). Subsequent application of IS-10 in pathogen-infested soil (T5) improved germination to 87.50% and slightly increased root biomass. Notably, the combined application of IS-10 and gum acacia (T6) significantly enhanced dry shoot weight to 126.66 mg plant⁻¹, representing a 63.4% increase over the pathogen-treated control (T4), while dry root weight increased to 16.50 mg plant⁻¹. Overall, the beneficial effects of *B. cabrialesii* IS-10 were more pronounced in the cultivar Vaibhav than in JG-62, suggesting cultivar-dependent responses to bacterial inoculation and carrier-assisted delivery (Table 4, Supplementary Figure 4).

### Disease severity and incidence after 30 days

**Fig. 5:**
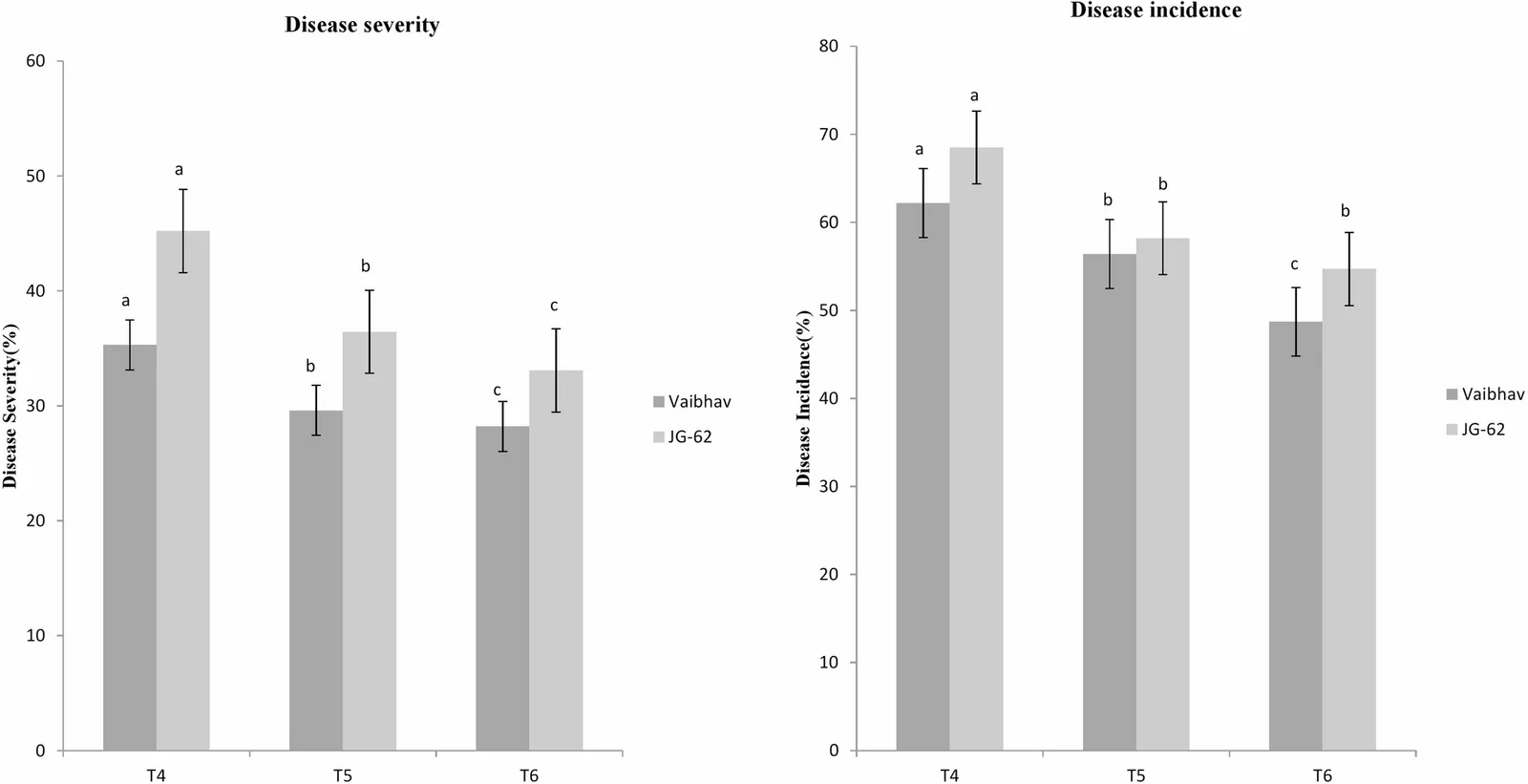
Effect of *B. cabrialesii* IS-10 inoculation on disease severity and incidence at 30 days of chickpea varieties in T4: Sterilized soil + sterilized vermicompost (0.2%) + *F.oxysporum *f. sp.*ciceris* ,T5: Sterilized soil + sterilized vermicompost (0.2%) + *F. oxysporum* f. sp. *ciceris* + B. cabrialesii (1mL of 10^8^ CFU/mL), T6: Sterilized soil + sterilized vermicompost (0.2%) + *F.oxysporum* f. sp. *ciceris*+ sterilized gum acacia (0.02%)+*B. cabrialesii*(1mL of 10^8^CFU/mL); Mean ± SD; Mean values on each bar with the different letters a,b,c etc showed the significant difference between each treatments by Duncan multiple comparison test (*P* ≤ 0.05)

No disease was observed in the first three treatments (T1-T3) where FOC was absent. In Treatment 4, which involved sterilized soil, sterilized vermicompost (0.2%), and FOC, the disease severity was 45.21% in the JG-62 cultivar and 35.3% in the Vaibhav cultivar. In Treatment 5, the T4 plus *Bacillus cabrialesii* IS-10, the disease severity decreased to 36.44% (a 19.3% reduction from T4) in JG-62 and to 29.6% (a 16.14% reduction from T4) in the Vaibhav cultivar. In Treatment 6, which included sterilized soil, sterilized vermicompost, FOC, sterilized gum acacia, and *B. cabrialesii*, the disease severity further decreased to 33.07% (a 26.85% reduction from T4) in the JG-62 cultivar and to 28.21% (a 20.08% reduction from T4) in the Vaibhav cultivar (Fig. 5).

The disease incidence in the JG-62 cultivar for Treatment 4 was 68.5%, compared to 62.2% in the Vaibhav cultivar. In Treatment 5, the disease incidence was 56.4% (a 9.32% decrease from T4) in Vaibhav and 58.2% (a 15.03% decrease from T4) in JG-62. For Treatment 6, JG-62 had a disease incidence of 54.7% (a 20.1% decrease from T4), while Vaibhav’s incidence was 48.7% (a 21.7% decrease from T4) (Fig. 5). Treatments that included FOC (T4) consistently exhibited higher disease severity and incidence percentages in both varieties compared to treatments without the pathogen. Overall, both disease severity and incidence were greater in JG-62 compared to Vaibhav. This variability may be attributed to Vaibhav’s moderate resistance in contrast to the disease-susceptible nature of JG-62.

### Bacterial population, soil dehydrogenase activity, and leaf chlorophyll content in chickpea cultivars

In both chickpea cultivars, bacterial population, soil dehydrogenase activity (DHA), and leaf chlorophyll content generally increased from the pre-flowering stage (30 days) to the flowering stage (45 days), particularly in treatments inoculated with *B. cabrialesii* IS-10 (Tables 5 and 6). The highest values for these parameters were consistently observed in treatments receiving *B. cabrialesii* IS-10 in combination with sterilized gum acacia (T3) or in conjunction with gum acacia under pathogen challenge (T6).

**Table 5:** Effect of *Bacillus cabrialesii* IS-10 inoculation on rhizospheric bacterial population, soil dehydrogenase activity, and leaf chlorophyll content at pre-flowering (30 days) and flowering (45 days) stages of chickpea cultivar Vaibhav (RG 2918) under non-challenged and *Fusarium oxysporum* f. sp. *ciceris*-challenged conditions

Treatments	Pre-flowering stage (30 days)			At flowering stage (45 days)		
	Bacterial population (CFU /g soil)	Soil dehydrogenase activity (pKat/ g soil)	Leaf chlorophyll content (mg/g leaf fresh weight)	Bacterial population (CFU /g soil)	Soil dehydrogenase activity (pKat/ g soil)	Leaf chlorophyll content (mg/g leaf fresh weight)
T 1	1.2 × 10^2^ (2.09 ± 0.05^d^)	28 0.18 ± 3.99^f^	1.613 ± 0.31^e^	1.8 × 10^2^ (2.25 ± 0.39^d^)	35.23 ± 4.03^f^	2.35 ± 0.21^d^
T 2	6.7 × 10 ^6^ (6.83 ± 0.43^b^)	37.9 ± 2.28^e^	1.930 ± 0.29^c^	3.7 × 10^7^ (7.57 ± 0.44^b^)	59.9 ± 2.51^d^	2.79 ± 0.11^b^
T 3	7.8 × 10^7^ (7.89 ± 0.61^a^)	58.75 ± 2.02^b^	2.340 ± 0.19^a^	8.9 × 10^8^ (8.95 ± 0.33^a^)	68.75 ± 5.16^c^	2.97 ± 0.18^a^
T 4	1.5 × 10^3^ (3.16 ± 0.24^c^)	41.13 ± 3.93^d^	1.539 ± 0.42^f^	0.5 × 10^4^ (4.70 ± 1.18^c^)	47.13 ± 3.07^e^	1.32 ± 0.20^f^
T 5	4.9 × 10^6^ (6.69 ± 0.67^b^)	57.58 ± 2.45^c^	1.860 ± 0.18^d^	4.9 × 10^6^ (6.69 ± 0.50^b^)	76.58 ± 2.99^b^	1.76 ± 0.35^e^
T 6	6.2 × 10^7^ (7.79 ± 0.81^a^)	67.78 ± 3.25^a^	2.010 ± 0.05^b^	5.4 × 10^8^ (8.73 ± 0.29^a^)	85.78 ± 3.06^a^	2.40 ± 0.24^c^
LSD (*p* ≤ 0.05)	1.39	4.6	0.301	2.81	5.3	0.34

Bacterial population values in parentheses are log₁₀-transformed. Treatment descriptions (T1–T6) are identical to those described in Table 4. Values are presented as mean ± SD (*n* = 3). Means within a column followed by different lowercase letters differ significantly according to Duncan’s Multiple Range Test (DMRT) at *p* ≤ 0.05

**Table 6:** Effect of *Bacillus cabrialesii* IS-10 inoculation on rhizospheric bacterial population, soil dehydrogenase activity, and leaf chlorophyll content at pre-flowering (30 days) and flowering (45 days) stages of chickpea cultivar JG-62 under non-challenged and *Fusarium oxysporum* f. sp. *ciceris*-challenged conditions

Treatments	Pre-flowering stage (30 days)			At flowering stage (45 days)		
	Bacterial population (CFU /g soil)	Soil dehydrogenase activity (pKat/ gsoil)	Leaf chlorophyll content (mg/g leaf)	Bacterial population (CFU /g soil)	Soil dehydrogenase activity (pKat/ g soil)	Leaf chlorophyll content (mg/g leaf)
T 1	2.5 × 10^2^ (2.40 ± 0.14c)	21.23 ± 2.54^f^	1.53 ± 0.13^c^	1.1 × 10^2^ (2.041 ± 0.39^d^)	25.11 ± 2.49^f^	1.872 ± 0.32^d^
T 2	4.3 × 10 ^6^ (6.61 ± 0.51b)	27.31 ± 3.00^d^	1.83 ± 0.03^a^	2.1 × 10 ^6^ (6.322 ± 0.33^b^)	32.5 ± 3.00^e^	2.343 ± 0.36^b^
T 3	8.1 × 10^8^ (8.86 ± 0.69a)	42.31 ± 1.89^a^	1.84 ± 0.09^a^	1.8 × 10^8^ (8.255 ± 0.71^a^)	74.38 ± 2.47^a^	2.531 ± 0.18^a^
T 4	0.7 × 10^3^ (2.85 ± 0.36c)	24.43 ± 1.88^e^	1.43 ± 0.12^d^	1.3 × 10^3^ (3.113 ± 0.55^c^)	43.29 ± 2.11^d^	1.523 ± 0.35^e^
T 5	3.1 × 10^6^ (6.50 ± 0.81c)	30.43 ± 1.25^c^	1.53 ± 0.11^c^	4.2 × 10^7^ (6.491 ± 0.26^b^)	49.6 ± 1.85^c^	2.321 ± 0.17^c^
T 6	4.3 × 10^6^ (6.61 ± 0.51c)	36.31 ± 1.93^b^	1.67 ± 0.04^b^	6.1 × 10^7^ (7.785 ± 0.88^a^)	52.26 ± 2.79^b^	2.342 ± 0.27^b^
LSD (*p* ≤ 0.05)	1.79	3.226	0.141	1.09	3.717	0.425

Bacterial population values in parentheses are log₁₀-transformed. Treatment descriptions (T1–T6) are identical to those described in Table 4. Values are presented as mean ± SD (*n* = 3). Means within a column followed by different lowercase letters differ significantly according to Duncan’s Multiple Range Test (DMRT) at *p* ≤ 0.05

In the cultivar Vaibhav, the control treatment (T1) recorded a bacterial population of 1.2 × 10² CFU g⁻¹ soil, soil DHA of 28.18 pKat g⁻¹ soil, and leaf chlorophyll content of 1.613 mg g⁻¹ fresh leaf weight at the pre-flowering stage. At flowering, these values increased slightly to 1.8 × 10² CFU g⁻¹ soil, 35.23 pKat g⁻¹ soil, and 2.35 mg g⁻¹ fresh leaf weight, respectively. Inoculation with *B. cabrialesii* IS-10 significantly enhanced all three parameters. The highest bacterial population was recorded in T3, reaching 7.8 × 10⁷ and 8.9 × 10⁸ CFU g⁻¹ soil at pre-flowering and flowering stages, respectively. Correspondingly, soil DHA increased to 58.75 and 68.75 pKat g⁻¹ soil, while leaf chlorophyll content increased to 2.34 and 2.97 mg g⁻¹ fresh leaf weight at the respective stages. Pathogen inoculation (T4) reduced chlorophyll content and soil biological activity; however, application of *B. cabrialesii* IS-10, particularly in combination with gum acacia (T6), effectively mitigated these adverse effects, resulting in the highest soil DHA (67.78 and 85.78 pKat g⁻¹ soil) and substantial chlorophyll accumulation (2.01 and 2.40 mg g⁻¹ fresh leaf weight) among the pathogen-challenged treatments.

A similar trend was observed in the susceptible cultivar JG-62, although the magnitude of response was generally lower than that observed in Vaibhav. The control treatment exhibited bacterial populations of 2.5 × 10² and 1.1 × 10² CFU g⁻¹ soil at pre-flowering and flowering stages, respectively, with corresponding soil DHA values of 21.23 and 25.11 pKat g⁻¹ soil. Inoculation with *B. cabrialesii* IS-10 significantly enhanced bacterial populations, soil DHA, and chlorophyll content, with the greatest improvements recorded in T3. At flowering, T3 exhibited a bacterial population of 1.8 × 10⁸ CFU g⁻¹ soil, soil DHA of 74.38 pKat g⁻¹ soil, and chlorophyll content of 2.531 mg g⁻¹ leaf tissue. Under pathogen pressure, T6 recorded significantly greater bacterial populations (6.1 × 10⁷ CFU g⁻¹ soil), soil DHA (52.26 pKat g⁻¹ soil), and chlorophyll content (2.342 mg g⁻¹ leaf tissue) than the pathogen-only treatment (T4), demonstrating the protective effect of *B. cabrialesii* IS-10.

Overall, inoculation with *B. cabrialesii* IS-10, particularly when combined with sterilized gum acacia, significantly increased bacter

ial establishment in the rhizosphere, enhanced soil biological activity, and improved leaf chlorophyll content in both chickpea cultivars. These beneficial effects were more pronounced in Vaibhav than in JG-62, indicating cultivar-dependent differences in responsiveness to bacterial inoculation under both healthy and pathogen-challenged conditions (Tables 5 and 6).

### Soluble protein content in the leaves and stems of chickpea cultivars

**Fig. 6:**
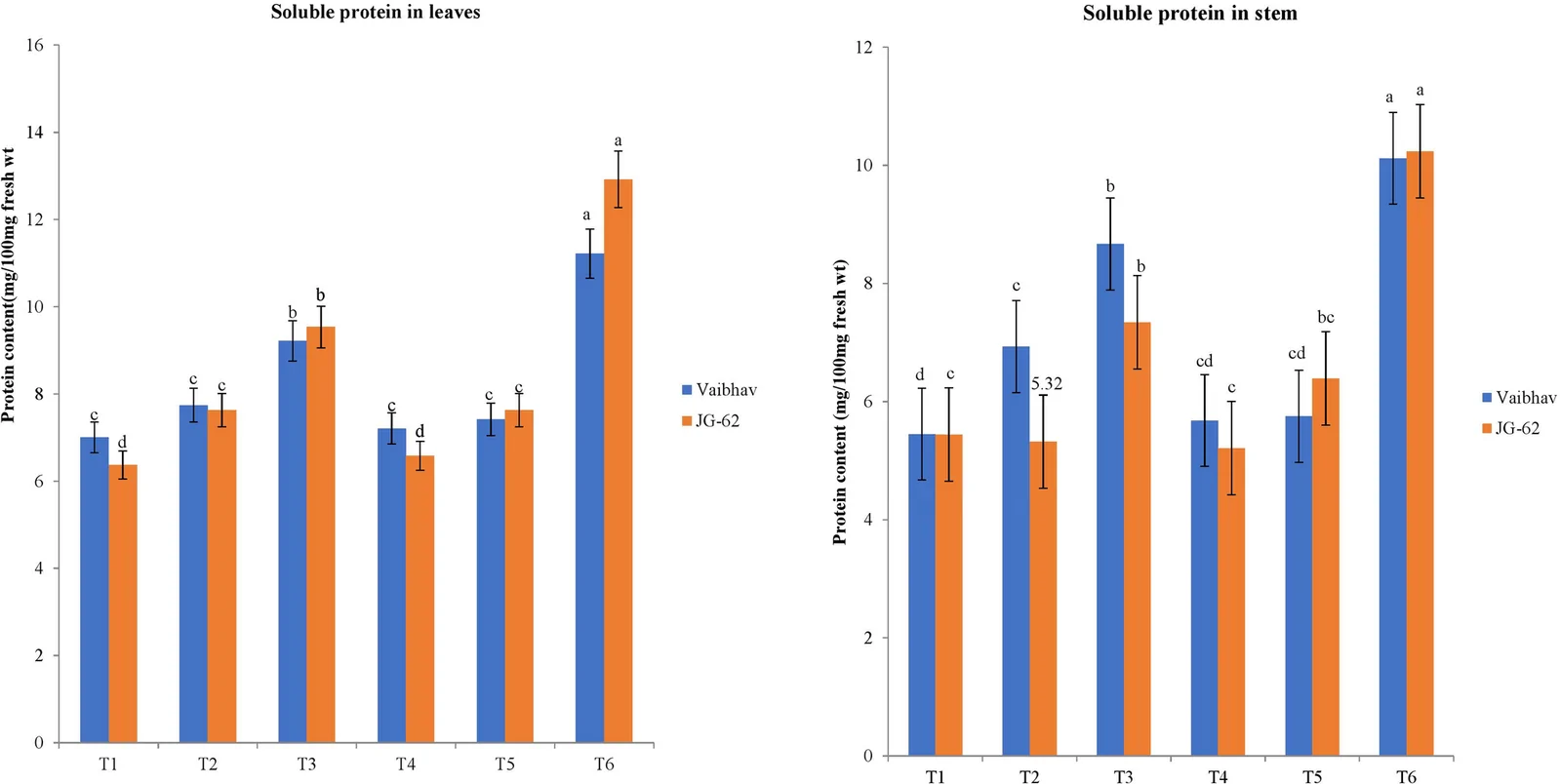
Effect of *B. cabrialesii* IS-10 inoculation on soluble protein content in leaf and stem of chickpea varieties in T1: Sterilized Soil + Sterilized vermicompost alone (5 ton/ha or 0.2%): Control, T2: Sterilized soil + sterilized vermicompost (0.2%) + *B. cabrialesii* (1mL of 10^8^ CFU/mL), T3: Sterilized soil + sterilized vermicompost (0.2%) +sterilized gum acacia (0.02% + *B. cabrialesii* (1mL of 10^8^ CFU/mL), T4: Sterilized soil + sterilized vermicompost + *F. oxysporum* f. sp. *ciceris*, T5: Sterilized soil + sterilized vermicompost (0.2%) + F. oxysporum f. sp. ciceris+ *B. cabrialesii* (1mL of 10^8^ CFU/mL), T6: Sterilized soil + sterilized vermicompost (0.2%) + *F. oxysporum* f. sp. *ciceris*+ sterilized gum acacia (0.02%)+ *B. cabrialesii* (1mL of 10^8^CFU/mL); Mean ± SD; Mean values on each bar with the different letters a,b,c etc showed the significant difference between each treatments by Duncan multiple comparison test (P ≤ 0.05)

The protein content in the leaves was generally higher than in the stems for both cultivars, regardless of inoculation. For leaf protein, the inoculation of strain IS-10 increased the protein content in both cultivars in the absence of pathogen challenge. However, when faced with a pathogen challenge, the increase in protein was more pronounced in the JG-62 variety, particularly when strain IS-10 was inoculated along with vermicompost and gum acacia. Inoculation with strain IS-10 significantly increased the protein content in the stems of both cultivars without FOC challenge, but this enhancement was more pronounced in JG-62 when challenged with the FOC pathogen (Fig. 6).

### Defence-related enzyme activities in chickpea cultivars

**Fig. 7:**
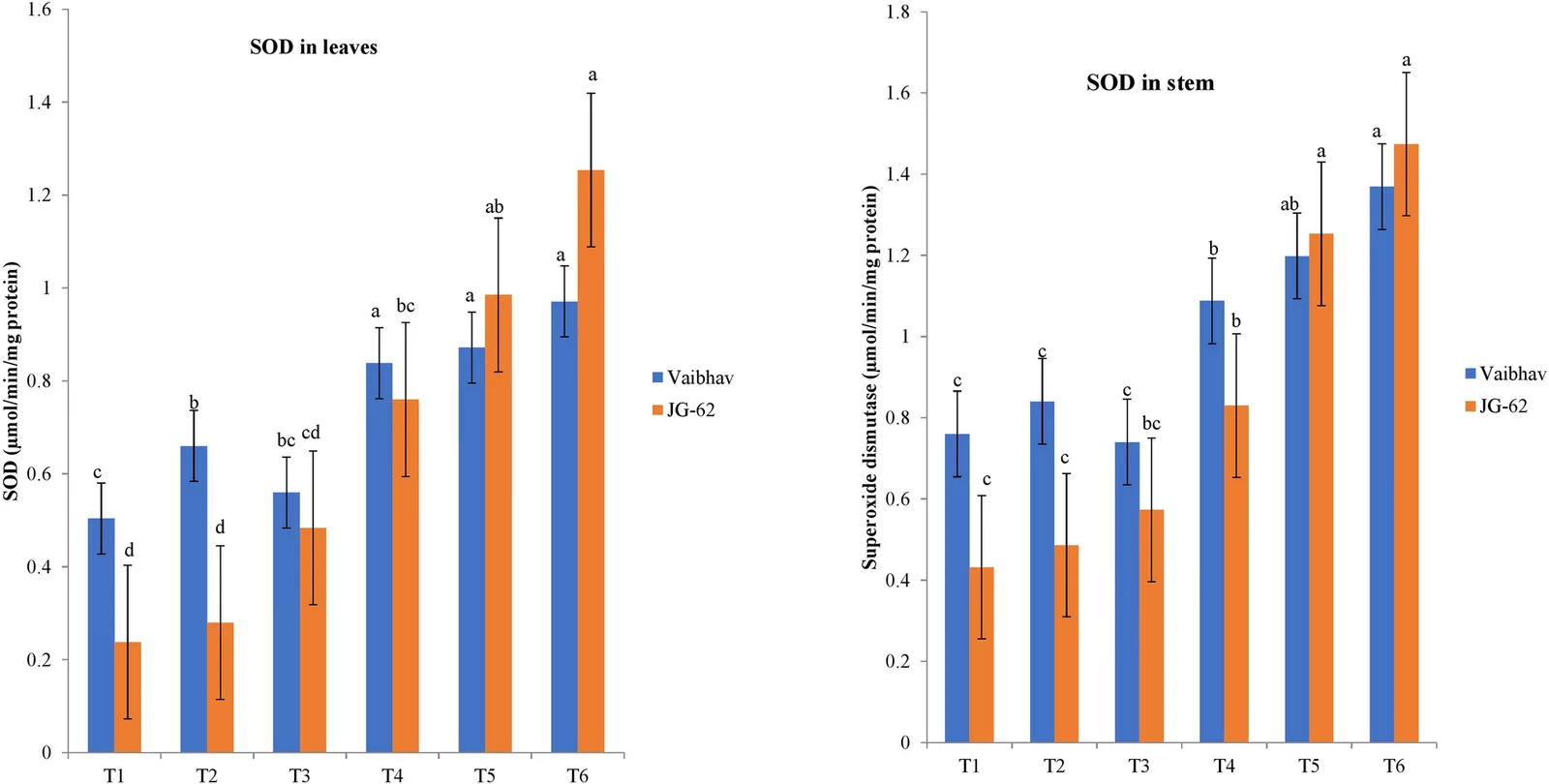
Effect of *B. cabrialesii* IS-10 inoculation on superoxide dismutase activity in leaf and stem of chickpea varieties in T1: Sterilized Soil + sterilized vermicompost alone (5 ton/ha or 0.2%): Control, T2: Sterilized soil + sterilized vermicompost (0.2%) + B. cabrialesii (1mL of 10^8^ CFU/mL), T3: Sterilized soil + sterilized vermicompost (0.2%) +sterilized gum acacia (0.02% + *B. cabrialesii* (1mL of 10^8^ CFU/mL), T4:Sterilized soil + sterilized vermicompost + *F. oxysporum* f. sp. *ciceris*, T5:Sterilized soil + sterilized vermicompost (0.2%) + *F. oxysporum* f. sp. *ciceris*+ *B. cabrialesii* (1mL of 10^8^ CFU/mL), T6: Sterilized soil + sterilized vermicompost(0.2%)+ *F. oxysporum* f. sp. *ciceris*+ sterilized gum acacia (0.02%) +*B. cabrialesii* (1mL of 10^8^ CFU/mL); Mean ± SD; Mean values on each bar with the different letters a,b,c etc showed the significant difference between each treatments by Duncan multiple comparison test (*P* ≤ 0.05)

**Fig. 8:**
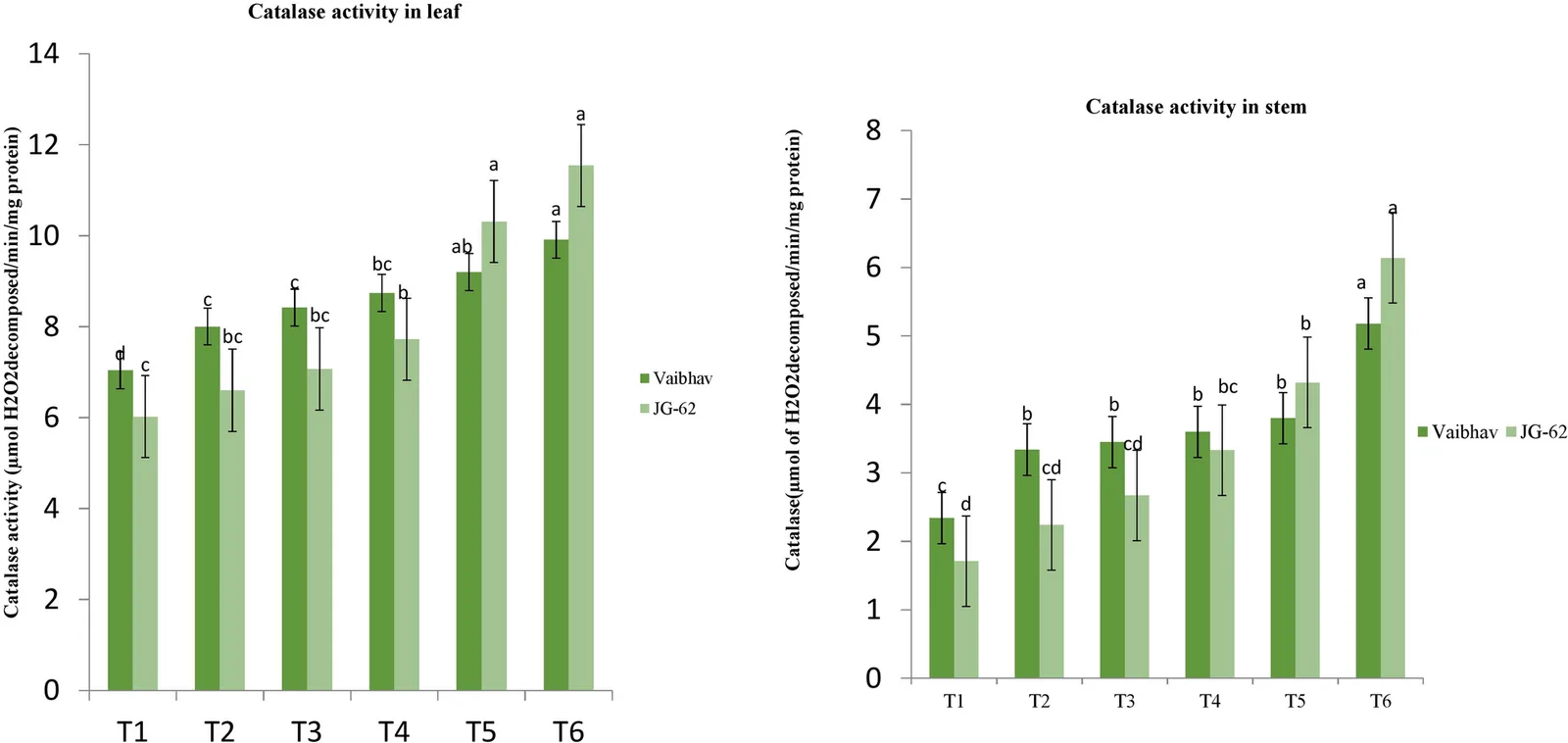
Effect of *B. cabrialesii* IS-10 inoculation on catalase activity in leaf and stem of chickpea varieties in T1:Sterilized Soil + sterilized vermicompost alone (5 ton/ha or 0.2%): Control, T2: Sterilized soil + sterilized vermicompost(0.2%)+ *B. cabrialesii* (1mL of 10^8^ CFU/mL), T3:Sterilized soil + Sterilized vermicompost (0.2%) + sterilized gum acacia (0.02%) + *B. cabrialesii* (1mL of 10^8^ CFU/mL), T4: Sterilized soil + sterilized vermicompost(0.2%) + *F. oxysporum* f. sp. *ciceris *,T5: Sterilized soil + sterilized vermicompost + *F. oxysporum* f. sp. *ciceris*+ *B. cabrialesii* (1mL of 10^8^ CFU/mL), T6: Sterilized soil + sterilized vermicompost (0.2%) + *F. oxysporum* f. sp. *ciceris*+ sterilized gum acacia (0.02%) +*B. cabrialesii* (1mL of 10^8^ CFU/mL); Mean ± SD; Mean values on each bar with the different letters a,b,c etc showed the significant difference between each treatments by Duncan multiple comparison test (*P* ≤ 0.05)

The activities of peroxidase (POD) and phenylalanine ammonia-lyase (PAL), two important defense-related enzymes, were significantly influenced by *B. cabrialesii* IS-10 inoculation and pathogen challenge in both chickpea cultivars (Table 7). In general, POD activity was consistently higher in stem tissues than in leaves across all treatments and cultivars. In general, SOD level in the leaves and stems of the Vaibhav cultivar were higher than those of JG-62, regardless of the inoculation with strain IS-10 without the presence of FOC (Fig. 7). SOD activity peaked in the leaves and stems of JG-62 when inoculated with strain IS-10 along with vermicompost and gum acacia after being pre-challenged with FOC. This suggests a higher induction of SOD in the more susceptible JG-62 cultivar (Fig. 7). Catalase activity was consistently observed to be higher in the leaves compared to the stems across all treatments and varieties (Fig. 8). In the absence of FOC, catalase activity in both the leaves and stems was greater in Vaibhav cultivar upon inoculation with strain IS-10 combined with vermicompost or gum acacia, compared to JG-62 cultivar. However, in the presence of FOC, JG-62 cultivar exhibited higher catalase levels than Vaibhav, indicating greater induction of this defense enzyme in the susceptible cultivar when inoculated with strain IS-10 (Fig. 8).

**Table 7:** Effect of *Bacillus cabrialesii* IS-10 inoculation on defense-related enzyme activities [peroxidase (POD) and phenylalanine ammonia-lyase (PAL)] in chickpea cultivars Vaibhav (RG 2918) and JG-62 under non-challenged and *Fusarium oxysporum* f. sp. *ciceris*-challenged conditions

Treatments	Vaibhav (RG 2918)				JG-62			
	Peroxidase (µmol H_2_O_2_reduced /min/mg protein)		Phenylalanine ammonia lyase (*n* mole cinnamic acid release/ h^/^ /g.fresh wt)		Peroxidase (µmol H_2_O_2_reduced /min/mg protein)		Phenylalanine ammonia lyase (*n* mole cinnamic acid release/ h^/^ /g. fresh wt)	
	Leaf	Stem	Leaf	Stem	Leaf	Stem	Leaf	Stem
T 1	0.568 ± 0.12^f^	0.978 ± 0.04^e^	69.92 ± 3.8^f^	101.38 ± 7.38^f^	0.525 ± 0.12^d^	0.925 ± 0.09^e^	69.19 ± 2.1^f^	100.54 ± 6.36^e^
T 2	0.771 ± 0.09^d^	1.181 ± 0.13c	74.32 ± 3.2^e^	107.9 ± 5.21^e^	0.669 ± 0.06^c^	1.079 ± 0.53^d^	71.90 ± 4.1^e^	104.25 ± 3.11^d^
T 3	0.643 ± 0.45^e^	1.053 ± 0.87^d^	77.52 ± 5.9^d^	112.4 ± 7.77^d^	0.510 ± 0.71^d^	0.92 ± 0.61^e^	73.42 ± 3.21^d^	106.45 ± 6.32^d^
T 4	0.937 ± 0.33^c^	0.347 ± 0.29^f^	91.90 ± 4.7^c^	128.18 ± 6.69c	0.909 ± 0.81^b^	1.319 ± 0.52^c^	88.84 ± 3.24^c^	121.59 ± 5.4^c^
T 5	1.176 ± 0.62^b^	1.586 ± 0.23^b^	95.86 ± 4.6^b^	133.25 ± 5.35^b^	0.255 ± 0.26^c^	1.665 ± 0.32^b^	99.7 ± 4.2 6^b^	144.56 ± 1.43^b^
T 6	1.436 ± 0.31^a^	1.846 ± 0.23^a^	110.94 ± 5.6^a^	160.30 ± 11.01	1.638 ± 0.38^a^	2.989 ± 0.72^a^	114.5 ± 6.51^a^	165.86 ± 9.74^a^
LSD (*p* ≤ 0.05)	0.107	0.269	4.2	8.08^a^	0.150	0.145	8.5	2.9

Treatment descriptions (T1–T6) are identical to those described in Table 4. Values are expressed as mean ± SD (*n* = 3). Means within a column followed by different lowercase letters differ significantly according to Duncan’s Multiple Range Test (DMRT) at *P* ≤ 0.05. POD activity is expressed as µmol H₂O₂ reduced min⁻¹ mg⁻¹ protein, whereas PAL activity is expressed as nmoles cinnamic acid released h⁻¹ g⁻¹ fresh weight

In the resistant cultivar Vaibhav (RG 2918), the control treatment (T1) recorded POD activities of 0.568 and 0.978 µmol H₂O₂ reduced min⁻¹ mg⁻¹ protein in leaves and stems, respectively. Inoculation with *B. cabrialesii* IS-10 alone (T2) or in combination with gum acacia (T3) resulted in moderate increases in POD activity. Pathogen challenge with *F. oxysporum* f. sp. *ciceris* (FOC) (T4) markedly enhanced POD activity, particularly in leaves (0.937 µmol H₂O₂ reduced min⁻¹ mg⁻¹ protein). The highest POD activities were observed in T6 (FOC + gum acacia + *B. cabrialesii* IS-10), reaching 1.436 and 1.846 µmol H₂O₂ reduced min⁻¹ mg⁻¹ protein in leaves and stems, respectively.

Similarly, PAL activity increased progressively in response to bacterial inoculation and pathogen challenge. In the control treatment, PAL activity was 69.92 and 101.38 nmoles cinnamic acid released h⁻¹ g⁻¹ fresh weight in leaves and stems, respectively. Application of *B. cabrialesii* IS-10 together with gum acacia (T3) increased PAL activity to 77.52 and 112.40 nmoles h⁻¹ g⁻¹ fresh weight in leaves and stems, respectively. Pathogen challenge alone (T4) further enhanced PAL activity, indicating activation of host defense responses. The highest PAL activities were recorded in T6, reaching 110.94 nmoles h⁻¹ g⁻¹ fresh weight in leaves and 160.30 nmoles h⁻¹ g⁻¹ fresh weight in stems, representing increases of 20.7% and 25.1%, respectively, over the pathogen-only treatment (T4).

A similar pattern was observed in the susceptible cultivar JG-62. The control treatment recorded POD activities of 0.525 and 0.925 µmol H₂O₂ reduced min⁻¹ mg⁻¹ protein and PAL activities of 69.19 and 100.54 nmoles cinnamic acid released h⁻¹ g⁻¹ fresh weight in leaves and stems, respectively. Inoculation with *B. cabrialesii* IS-10, particularly when combined with gum acacia, resulted in modest increases in both enzyme activities under non-challenged conditions. Following pathogen challenge, substantial induction of defense enzymes was observed. The highest POD activities were recorded in T6, reaching 1.638 and 2.989 µmol H₂O₂ reduced min⁻¹ mg⁻¹ protein in leaves and stems, respectively. Likewise, PAL activity increased to 114.50 and 165.86 nmoles cinnamic acid released h⁻¹ g⁻¹ fresh weight in leaves and stems, respectively, representing increases of 28.9% and 36.4% over the pathogen-only treatment (T4).

Overall, pathogen challenge stimulated both POD and PAL activities in the two cultivars, while inoculation with *B. cabrialesii* IS-10 further enhanced these defense responses. The combined application of *B. cabrialesii* IS-10 and gum acacia (T6) consistently produced the highest enzyme activities, indicating enhanced induction of host defense mechanisms and improved tolerance against Fusarium wilt. The magnitude of enzyme induction was generally greater in the susceptible cultivar JG-62 than in Vaibhav, suggesting a stronger biochemical response to pathogen stress and bacterial inoculation in the susceptible host genotype.

### Agronomic attributes at harvest of chickpea cultivars

At harvest (84 days), significant differences in plant growth and yield attributes were observed among treatments in both chickpea cultivars (Table 8). In general, inoculation with *B. cabrialesii* IS-10 improved plant growth and yield parameters, with the greatest benefits observed when the bacterial inoculant was applied in combination with sterilized gum acacia (T3) or under pathogen-challenged conditions together with gum acacia (T6).

**Table 8:** Effect of *Bacillus cabrialesii* IS-10 inoculation on growth and yield attributes at harvest (84 days after sowing) of chickpea cultivars Vaibhav (RG 2918) and JG-62 under non-challenged and *Fusarium oxysporum* f. sp. *ciceris*-challenged conditions

Treatments	Vaibhav (RG 2918)					JG-62				
	Plant height (cm)	Root weight (g/plant)	Pod numbers (per plant)	Straw yield (g/plant)	Grain yield (g/plant)	Plant height (cm)	Root weight (g/plant)	Pod numbers (per plant)	Straw yield (g/plant)	Grain yield (g/plant)
T 1	28.9 ± 1.23^de^	0.203 ± 0.01^e^	13 ± 1.83^c^	3.264 ± 0.08^c^	2.5 ± 0.63^c^	28.7 ± 1.59^bc^	0.22 ± 0.019^b^	8 ± 1.15^d^	2.3 ± 0.26^bc^	1.55 ± 0.33^cd^
T 2	29.85 ± 2.03^cd^	0.28 ± 0.01^c^	20 ± 1.15^a^	4.864 ± 0.29^ab^	3.8 ± 0.27^ab^	28.7 ± 0.97^bc^	0.27 ± 0.021^ab^	14 ± 0.82^bc^	2.8 ± 0.08^b^	2.31 ± 0.23^b^
T 3	33.4 ± 1.77^a^	0.62 ± 0.042^a^	22 ± 1.63^a^	5.36 ± 0.14^a^	4.7 ± 0.50^a^	31.33 ± 1.75^a^	0.25 ± 0.033^ab^	18 ± 1.15^a^	3.8 ± 0.15^a^	3.12 ± 0.41^a^
T 4	27.77 ± 1.93^e^	0.21 ± 0.03^de^	10 ± 0.82^d^	2.241 ± 0.14^d^	1.8 ± 0.18^c^	27.02 ± 1.69^c^	0.27 ± 0.016^ab^	7 ± 0.58^d^	1.9 ± 0.08^c^	1.21 ± 0.09^d^
T 5	30.28 ± 2.39^bc^	0.26 ± 0.03^cb^	17 ± 0.82^b^	4.608 ± 0.72^b^	3.7 ± 0.24^b^	27.68 ± 1.85^c^	0.26 ± 0.016^ab^	12 ± 1.83^c^	2.4 ± 0.18^bc^	2.05 ± 0.02^bc^
T 6	31.84 ± 1.37^ab^	0.465 ± 0.029^a^	20 ± 1.83^a^	4.864 ± 0.17^a^	4.2 ± 0.16^ab^	30.33 ± 2.36^ab^	0.32 ± 0.014^a^	15 ± 1.41^b^	3.0 ± 0.18^ab^	2.46 ± 0.07^ab^
LSD (*p* ≤ 0.05)	2.74	0.042	2.11	0.47	0.55	2.62	0.031	1.83	0.25	0.36

Treatment descriptions (T1–T6) are identical to those described in Table 4. Values are presented as mean ± SD (*n* = 3). Means within a column followed by different lowercase letters differ significantly according to Duncan’s Multiple Range Test (DMRT) at *P* ≤ 0.05

In the moderately resistant cultivar Vaibhav (RG 2918), the highest plant height (33.4 cm), root weight (0.62 g plant⁻¹), pod number (22 pods plant⁻¹), straw yield (5.36 g plant⁻¹), and grain yield (4.7 g plant⁻¹) were recorded in T3, comprising sterilized vermicompost, gum acacia, and *B. cabrialesii* IS-10. Compared with the control (T1), this treatment increased plant height by 15.6%, root weight by 205.4%, pod number by 69.2%, straw yield by 64.2%, and grain yield by 88.0%. Pathogen inoculation alone (T4) significantly reduced all agronomic attributes, resulting in the lowest values for plant height (27.77 cm), pod number (10 pods plant⁻¹), straw yield (2.24 g plant⁻¹), and grain yield (1.8 g plant⁻¹). However, the introduction of *B. cabrialesii* IS-10 in pathogen-infested soil improved plant growth and productivity. The combined application of IS-10 and gum acacia (T6) increased plant height to 31.84 cm, pod number to 20 pods plant⁻¹, straw yield to 4.86 g plant⁻¹, and grain yield to 4.2 g plant⁻¹, demonstrating substantial mitigation of Fusarium wilt-induced yield losses.

A similar trend was observed in the susceptible cultivar JG-62. The highest plant height (31.33 cm), pod number (18 pods plant⁻¹), straw yield (3.8 g plant⁻¹), and grain yield (3.12 g plant⁻¹) were recorded in T3. Relative to the control, this treatment increased pod production by 125%, straw yield by 65.2%, and grain yield by 101.3%. In contrast, pathogen inoculation alone (T4) caused marked reductions in all growth and yield parameters, with grain yield declining to 1.21 g plant⁻¹. Application of *B. cabrialesii* IS-10 under pathogen challenge partially alleviated these adverse effects, while the combination of IS-10 and gum acacia (T6) produced the greatest recovery, resulting in a plant height of 30.33 cm, root weight of 0.32 g plant⁻¹, 15 pods plant⁻¹, straw yield of 3.0 g plant⁻¹, and grain yield of 2.46 g plant⁻¹.

Overall, inoculation with *B. cabrialesii* IS-10 significantly improved growth and yield-related traits in both cultivars, particularly when delivered through sterilized gum acacia. The beneficial effects were generally more pronounced in the cultivar Vaibhav than in JG-62, especially under pathogen-challenged conditions, reflecting the greater capacity of the moderately resistant cultivar to respond to bacterial inoculation and withstand Fusarium wilt stress.

## Discussion

The genus *Bacillus* is widely recognized as a leading bacterium for promoting plant growth and suppressing diseases in sustainable agriculture. Plant growth promotion occurs through the production of ammonium, siderophores, and growth hormones. Meanwhile, the biological control of soil-borne pathogens is achieved through the production of hydrolytic enzymes and secondary metabolites, such as antimicrobial peptides (AMPs). In addition to these mechanisms, competition for nutrients and space, as well as the induction of defense responses, have also been reported to contribute to plant growth promotion and disease control [37]. In this study, we present two strains of *B. cabrialesii*, which were isolated from the rhizosphere of chickpeas cultivated in the Raipur and Bemetra districts of Chhattisgarh state, India. To our knowledge, this is the first report of the occurrence of *B. cabrialesii* in the state of Chhattisgarh. The initial isolation of *B. cabrialesii* TE3^T^ was documented from Yaqui Valley, Mexico [38], and it has since been reported from various environments and crops around the world, including India. Notably, these include: (1) strains BH5 and BH6 from tomatoes in the Netherlands [39], (2) strain BKI 3 from rice in Telangana state, India [40], (3) strain FA26 from the soil rhizosphere of rice cultivated in the Sindh region of Pakistan [41], (4) strain F26 isolated from Soumbala, Burkina Faso [42], (5) strain/accession MZ342760 isolated from *Eisenia foetida*, a worm involved in vermicomposting in Pakistan, (6) strain SM-93 isolated from wheat in Pakistan [43], and (7) strains NOK78 and NOK82 isolated from wheat cultivated in India [13]. The occurrence of strains of this bacterium with varied traits in various countries suggests its ubiquitous nature.

Two strains, IS-10 and BATS-13, of *B. cabrialesii* were found to have more or less morphologically similar colonies, with the exception of a lobate margin observed in BATS-13. Both strains tested positive for citrate utilization, catalase, oxidase, and nitrate reduction, but tested negative for indole production. The characteristics of these strains closely matched those of the type strain TE3^T^. *Bacillus* species are abundant in the rhizosphere, with *B. subtilis*,* B. megaterium*,* B. mucilaginosus*,* B. pumilus*,* B. licheniformis*, and *B. cabrialesii* being the most predominant and widely studied. These species are known for their ability to colonize plant roots, solubilize potassium and phosphorus, promote root development and dry matter accumulation, and enhance overall plant yield [44]. The *B. cabrialesii* strains studied in this investigation exhibited several plant growth-promoting (PGP) traits. These traits include the production of hydrolytic enzymes, siderophores, indole-3-acetic acid (IAA), ammonium, and the ability to solubilize phosphate and zinc. These findings are consistent with previous reports [12, 13]. *Bacillus* species produce IAA, which the most important function of phytohormone that regulates various growth-related processes in plants [10]. The strains MPSK109 and BH5 of *B. cabrialesii* produced IAA, while strain BH5 also produced ammonium [12, 39]. Additionally, TE3^T^, BH5, NOK78, and NOK82 strains of *B. cabrialesii* exhibited phosphate solubilization properties, supporting our findings that strains IS-10 and BATS-13 were also capable of solubilizing phosphate and zinc phosphate. However, zinc phosphate solubilization was evident only in the MPSK109 strain [13]. Additionally, the production of amylase, protease, and siderophores by the IS-10 and BATS-13 strains was supported by the findings from strains MPSK109, NOK82, and BH5, which produced all these compounds [13]. Notably, siderophores, which are small iron-chelating agents (500–1000 Daltons), sequester available iron, making it unavailable for fungal phytopathogens, thereby aiding in pathogen control [45]. Siderophores play a crucial role in making iron available for various biological processes in plants. Previous studies have demonstrated that strains of *Bacillus atrophaeus* have the potential to produce siderophores, which can contribute to the promotion of plant growth [46]. Additionally, hydrolytic enzymes such as cellulase, amylase, protease, and lipase, produced by *Bacillus* species, serve a dual purpose: they promote plant growth and help break down the cell walls of pathogens. Hydrolytic enzymes also provide energy to germinating plants by solubilizing starch, lipids, and proteins in the endosperm of seeds [47]. Additionally, these enzymes produced by *B. atrophaeus* TS1 inhibit *Fusarium graminearum* [48]. It has been reported that hydrolytic enzymes produced by *Bacillus* species play a crucial role in penetrating plant tissue and facilitating endophytic colonization within host plants [49]. Additionally, *B. cereus*,* B. amyloliquefaciens*, and *B. subtilis* subsp. *subtilis*, which are associated with pearl millet, have been shown to solubilize potassium and produce siderophores. This highlights their roles as growth promoters, nutrient mobilizers, and disease defenders [50]. Interestingly, our study found that certain strains stimulated the growth of *Fusarium oxysporum* f. sp. *ciceris* (FOC) through volatile compounds released by strain IS-10. This contrasts with other reports indicating that *B. cabrialesii* BH5 inhibited the growth of *Fusarium culmorum*,* Pythium ultimum*,* Botrytis cinerea*, and *Rhizoctonia solani* [39]. This divergence may be attributed to the release of stimulatory volatiles without hydrogen cyanide (HCN) by these bacterial strains. Thus, it can be inferred that *B. cabrialesii* strains can protect against phytopathogens and promote plant growth through various direct and indirect mechanisms.

PGPR reduce the effects of pathogens by combating phytopathogenic microorganisms through the production of fungicidal chemicals, siderophores, antibiotics, enzymes, etc. Lipopeptides (LPs) are a group of secondary metabolites produced by microorganisms that serve various biological functions, including acting as surface-active agents (surfactants) with cytotoxic or antibacterial properties [51]. Among the five types of lipopeptides produced by *Bacillus* species, surfactin, iturin, and fengycin are the three most significant families [52]. *Bacillus thuringiensis* CHGP12 is a promising strain known for its strong antagonistic activity against *Fusarium oxysporum* f. sp. *cubense* (FOC), inhibiting its growth by 3.45 cm. This effect is achieved through the production of lipopeptides such as fengycin, surfactin, iturin, bacillaene, bacillibactin, plantazolicin, and bacilysin, along with their growth-promoting potential [10]. Additionally, *Bacillus cabrialesii* BH5 has demonstrated antagonistic activity against *Fusarium culmorum*,* Botrytis cinerea*,* Pythium ultimum*, and *Rhizoctonia solani*, primarily by producing fengycin and surfactin [39]. In this study, strains IS-10 and BATS-13 of *B. cabrialesii* produced lipopeptides (surfactin and fengycin), which inhibited FOC by 48.34% and 42.82%, respectively, and similarly inhibited *Sclerotium rolfsii* by 44.05% and 40.75%, and *Macrophomina phaseolina* by 48.34% and 42.82%. UPLC-ESI-MS/MS analysis revealed characteristic ion peaks corresponding to surfactin and fengycin lipopeptides produced by strain IS-10. These metabolites are well known for their significant antifungal activity against several phytopathogens [10, 39]. In addition, the present study demonstrated that volatile compounds released by *B. cabrialesii* strains IS-10 and BATS-13 stimulated the growth of *Fusarium oxysporum* f. sp. *ciceris* (FOC), which contrasts with the findings of Zhou et al. [39], who reported inhibitory effects of volatile compounds produced by *Bacillus* species. The volatile-mediated stimulation of FOC growth observed in the present investigation may reflect strain-specific metabolic interactions and environmental influences. Since volatile compounds were not chemically characterized in this study, definitive conclusions regarding the metabolites responsible for the stimulatory effect cannot be drawn. Therefore, further investigations involving GC-MS-based volatile profiling and environmental optimization studies will be necessary to elucidate the underlying mechanisms governing this interaction. Nevertheless, the antagonistic activity exhibited by *B. cabrialesii* strains IS-10 and BATS-13 against FOC may primarily be attributed to lipopeptide production and other unidentified biocontrol mechanisms rather than hydrogen cyanide (HCN) production or chitinase activity.

*Bacillus* spp. produces ammonia, lipopeptide surfactants, hydrolytic enzymes, and iron quenching activity. Together with the stimulation of defense-related enzymes, these factors strengthen the cell wall barrier and decrease pathogen colonization and migration. This indirectly indicates that *Bacillus* spp. has suppressive effects on pathogens, as further confirmed by reductions in disease incidence and severity [53]. Inoculation with *Bacillus velezensis* SM-39 and *B. cabrialesii* SM-93 was reported to significantly reduce the severity of Fusarium root rot in wheat by 42% to 62%, according to net house experiments conducted by Mulk et al. [43]. Similarly, Vanama et al. [54] performed glasshouse experiments that demonstrated a substantial decrease in stem rot disease in rice plants treated with a consortium of *B. cabrialesii* BIK3, *Trichoderma asperellum* TAIK1, *Pseudomonas putida* PIK1, and *P. otitidis* POPS, leading to a 55.92% reduction compared to the control. Our findings are consistent with those of Mulk et al. [43] and Vanama et al. [54], indicating that disease severity and incidence percentages were consistently higher in the presence of *Fusarium oxysporum* f. sp. *ciceris* (FOC) (T4) across both chickpea cultivars: JG-62, which is a susceptible variety, and Vaibhav, a moderately resistant cultivar. The introduction of the beneficial bacterial agent *B. cabrialesii* IS-10, in combination with FOC (T5), resulted in a significant reduction in both disease severity and incidence. The observed lower levels of disease severity and incidence with *B. cabrialesii* IS-10 suggest that this beneficial bacterium helps mitigate the impact of the disease. Moreover, the combination of sterilized gum acacia, *B. cabrialesii* IS-10, and *F. oxysporum* f. sp. *ciceris* (T6) further enhanced the reduction of disease severity and incidence in both chickpea cultivars. This synergistic effect implies that gum acacia enhances the action of *B. cabrialesii* IS-10, leading to a more significant decrease in disease severity and incidence. A comparison of disease severity and incidence percentages between the two chickpea cultivars shows that the moderately resistant cultivar, Vaibhav, consistently displayed lower disease severity and incidence compared to the susceptible cultivar, JG-62. This difference may be attributed to the inherent resistance of Vaibhav, allowing it to mount a stronger defense response against the pathogen.

Rhizobacteria that promote plant growth can trigger induced systemic resistance (ISR), a response that enhances the plant’s innate defenses against biotic threats. This is achieved through the increased production of enzymes, including catalase, peroxidase, superoxide dismutase (SOD), and phenylalanine ammonia-lyase (PAL) [55]. This study aimed to observe the accumulation of defense-related enzymes and superoxide radicals to evaluate the role of bacteria in inducing systemic resistance in chickpeas challenged with FOC. Reactive oxygen species (ROS), such as hydrogen peroxide (H₂O₂), superoxide (O₂⁻), singlet oxygen (¹O₂), and hydroxyl radicals (OH⁻), are generated as signaling molecules in plants under biotic stresses. The antioxidant systems in plants help regulate the levels of superoxide and other ROS. The reduced accumulation of superoxide in treatments inoculated with the strain IS-10 indicates protective effects against harmful superoxide levels during biotic stress conditions.

Bacteria stimulate the expression of PAL, SOD, peroxidase (PO), and catalase in the host plant, protection against pathogens. In treatments T5 (*B. cabrialesii* IS-10 and FOC) and T6 (sterilized gum acacia, *B. cabrialesii* IS-10, and FOC), the activity of PAL, SOD, PO, and catalase was significantly higher in IS-10 inoculated plants compared to the pathogen control (treatment 4). Similarly, Kumari and Khanna [56] found that chickpea challenged with FOC and treated with antagonistic rhizobacteria strains like Ps 45, Ba1a, and *Mesorhizobium* showed higher activities of PAL, SOD, peroxidase, and polyphenol oxidase (PPO) compared to the pathogen control. In a related study, Magashwaran et al. [57] found that inoculating chickpea plants with *Bacillus subtilis* TRO4 and *B. subtilis* CLO5 resulted in a reduction of superoxide accumulation. This treatment also enhanced the production of phenylalanine ammonia-lyase (PAL) and peroxidase (PO) in chickpea plants that were pre-challenged with *Fusarium oxysporum* f. sp. *ciceris* (FOC). Furthermore, applying a talc-based biofertilizer containing *Bacillus subtilis* to chickpea challenged with FOC significantly increased the levels of SOD, POX, and chlorophyll [58].

The variability in antagonistic efficiency among different *B. cabrialesii* strains may be influenced by ecological origin, rhizospheric adaptation, and host-associated selective pressures. Strain IS-10 was isolated from chickpea rhizosphere soil, which may have favored the evolution of niche-specific traits associated with chickpea root colonization and suppression of *Fusarium oxysporum* f. sp. *ciceris* (FOC). The strain was isolated and evaluated under the agroclimatic conditions of Raipur, Chhattisgarh, during the winter season, where maximum temperatures ranged from 25 to 30 °C and minimum temperatures ranged from 7 to 14 °C between 3 December 2022 and 4 March 2023 at the Research Farm, Raipur. Such environmental conditions may have contributed to the adaptation and functional efficiency of strain IS-10. In contrast, strains isolated from wheat, tomato, or non-rhizospheric environments may differ in metabolite production, colonization efficiency, and pathogen-target specificity. These environment–strain–function relationships are increasingly recognized as critical determinants of plant growth-promoting rhizobacteria (PGPR) performance under diverse agroecological conditions.

Phenylalanine ammonia-lyase (PAL) facilitates the formation of several compounds, including p-coumaric acid, cinnamic acid, caffeic acid, gallic acid, and ferulic acid, all of which contribute to the lignification and thickening of plant cell walls [59]. The phenylpropanoid pathway regulates various physiological processes within plant cells. It modulates the expression of key genes, leading to the induction of Induced Systemic Resistance (ISR) and Systemic Acquired Resistance (SAR) against biotic stresses in different plant species [59]. Our findings align with those of Singh et al. [60], who reported that inoculating plants with *Bacillus amyloliquefaciens* and other Plant Growth-Promoting Rhizobacteria (PGPR) activates defense-related pathways and upregulates specific enzymes over time. Generally, increased PAL activity is associated with inhibiting the growth of pathogenic fungi. This is achieved through the formation of phytoalexins, callose deposition, lignification of cell walls, production of antimicrobial secondary metabolites, and the expression of pathogenesis-related (PR) proteins [59].

In general, the levels of SOD, peroxidase, catalase, and PAL activity were found to be inherently higher in the Vaibhav cultivar, which is known for its moderate resistance to diseases, compared to the JG-62 cultivar without any inoculation. This indicates that resistance traits may be associated with antioxidant responses. Notably, the inclusion of *B. cabrialesii* IS-10 along with the FOC pathogen resulted in a substantial increase in SOD, peroxidase, catalase, and PAL activity in both varieties. This indicates that the beneficial bacteria played a crucial role in enhancing antioxidant defenses, even under pathogenic stress. Therefore, *B. cabrialesii* IS-10 may contribute to the overall resilience of chickpea plants against pathogenic infections by strengthening their antioxidant capabilities. The addition of gum acacia alongside *B. cabrialesii* IS-10 and the FOC pathogen further raised the levels of SOD, peroxidase, catalase, and PAL activity in both cultivars. Interestingly, while inoculation raised the levels of these enzymes in the Vaibhav cultivar, the increase was more pronounced in the JG-62 cultivar. This suggests that the beneficial bacteria may have a more significant influence on the susceptible cultivar, which inherently has lower antioxidant enzyme levels.

The rhizosphere is a chemically and biochemically complex environment that supports a diverse range of microbial communities. Several factors, both biotic and abiotic, influence the composition of these microbial communities in the rhizosphere. These factors include environmental conditions, soil physicochemical characteristics, the biological activities of plants, and the chemical signals emitted by both plants and rhizospheric soil bacteria [61].

In this study, the population of *Bacillus cabrialesii* IS-10 significantly increased in the rhizosphere of two chickpea cultivars, Vaibhav and JG-62, over time when inoculated with vermicompost and gum acacia. This increase was particularly noted in treatment 3, which correlated with high dehydrogenase activity. This increase in dehydrogenase activity, linked to the growing bacterial population in the rhizosphere, is supported by research conducted by Praveen-Kumar and Tarafdar [62], who found that dehydrogenase activity in soil is primarily attributable to bacteria rather than actinomycetes or fungi, especially in arid soils. Previous reports have shown that the richness and diversity of rhizosphere bacteria tend to increase after pre-inoculation with plant growth-promoting rhizobacteria (PGPR). For example, Romero-Silva and colleagues [63] discovered that inoculating a consortium of bacterial strains, including *B. cabrialesii* subsp. *cabrialesii* TE3^T^, subsp. tritici TSO2T, *B. paralicheniformis* TRQ65, *Bacillus* sp. FSQ1 and TE5, *B. subtilis* TSO22, and *Priestia megaterium* TRQ8 and TSO9 significantly affected fungal diversity across various crops at different phenological stages. In contrast, the bacterial diversity remained consistent across all crops.

In the non-challenged condition of this study, inoculation with *B. cabrialesii* IS-10 in combination with gum acacia enhanced the growth and yield of chickpea varieties, which is supported by findings from various researchers. This indicates that the promotion of plant growth in crops such as wheat, tomato, rice, and chilli pepper is attributed to various functional traits. These traits include the production of indoles, phosphate solubilization, and providing protection against pathogens [12, 40, 41]. Additionally, other *Bacillus* species have been found to enhance plant development through several direct mechanisms. These mechanisms include associative nitrogen fixation, nutrient solubilization (beyond just phosphate), siderophore production, phytohormone release, and the reduction of heavy metal toxicity [64].

## Conclusion

A significant discovery has revealed two strains, IS-10 and BATS-13, of *Bacillus cabrialesii* in the rhizosphere soil of chickpeas grown in Chhattisgarh state, India. This finding is significant as it marks the first documented occurrence of these beneficial strains in the region. Notably, *B. cabrialesii* IS-10 has exhibited remarkable plant growth-promoting capabilities, effective biocontrol mechanisms, and the production of valuable biosurfactants, such as surfactin and fengycin. These important attributes significantly help fight Fusarium wilt in chickpeas while enhancing plant growth and overall yield. This discovery not only highlights the potential of these strains to enhance agricultural practices but also underscores the importance of exploring local microbial resources for sustainable farming solutions.

Future studies involving multi-location field validation, molecular characterization of induced resistance pathways, and comparative evaluation with standard bioinoculants and fungicidal treatments will further strengthen the translational applicability of the present findings.

## Supplementary Information

Supplementary Material 1.

Supplementary Material 2.

Supplementary Material 3.

Supplementary Material 4.

Supplementary Material 5.

Supplementary Material 6.

## Data Availability

All data supporting the findings of this study are available within the paper and its Supplementary Information. The raw sequencing reads of the bacterial strains IS-10 and BATS-13 have been deposited in the NCBI database under accession numbers PQ795767 and PQ795772, respectively.
