# Supplementary material for: Inoculation of Bacillus cabrialesii modulates plant growth and wilt disease of chickpea cultivars pre-challenged with Fusarium oxysporum f. sp. ciceris
Source: BMC Microbiol. 2026 Jul 13;26:861. doi: 10.1186/s12866-026-05357-1 (PMC13637128; doi:10.1186/s12866-026-05357-1)
Supplement: Supplementary file 1 [file 12866_2026_5357_MOESM1_ESM.docx]

**Caption of Supplementary Figures**

**Supplementary Fig. 1**: Phylogenetic tree of *B.* Neighbor-Joining method, bootstrap: 1000, Evolutionary distance computed through Kimura-2, MEGA 11

**Supplementary Fig.2**: Plant growth promoting traits of *B.cabrialesii* strains (A) IAA production (B) Ammonia production, (C) Phosphate solubilization (D) & (E) Protease activity of *B.cabrialesii* IS-10 and *B.cabrialesii* BATS-13, (F)&(G)Siderophore activity of *B. cabrialesii* IS-10 and *B. cabrialesii* BATS-13, (H)&(I) Amylase activity of *B. cabrialesii* IS-10 and *B. cabrialesii* BATS-13, (J)&(K) Lipase activity of *B.cabrialesii* IS-10 and *B. cabrialesii* BATS-13,(L)&(M) Cellulase activity of *B. cabrialesii* IS-10 and *B. cabrialesii* BATS-13.

**Supplementary Fig 3**: Antagonistic activity of *B. cabrialesii* strains against *F. oxysporum* f.sp. *ciceris* and *S. rolfsii* (A)&(B) Antagonistic activity of *B. cabrialesii* IS-10 and *B.cabrialesii* BATS-13 against *F. oxysporum* f.sp. *ciceris*, (C)&(D) Antagonistic activity of *B. cabrialesii* IS-10and *B. cabrialesii* BATS-13 against *S. rolfsii*.

**Supplementary Fig 4**: Plant growth status of chickpea cv Vaibhav in different treatments. (T1-T6 – Treatments; V- Vaibhav cultivar)
