# Supplementary material for: Inoculation of Bacillus cabrialesii modulates plant growth and wilt disease of chickpea cultivars pre-challenged with Fusarium oxysporum f. sp. ciceris
Source: BMC Microbiol. 2026 Jul 13;26:861. doi: 10.1186/s12866-026-05357-1 (PMC13637128; doi:10.1186/s12866-026-05357-1)
Supplement: Supplementary file 2 [file 12866_2026_5357_MOESM5_ESM.docx]

**Supplementary Table 1**: Colony morphology of *B. cabrialesii* strains

| **Bacterial strains** | **Size** | **Shape** | **Margin** | **Elevation** | **Surface appearance** | **Pigmentation** |
| --- | --- | --- | --- | --- | --- | --- |
| **IS-10** | Medium | Irregular | Wavy | Raised | Rough | Off white |
| **BATS-13** | Medium | Irregular | Lobate | Raised | Rough | Off white |

**Supplementary Table 2**: Selected biochemical traits of *B.cabrialesii* strains

| **Biochemical traits** | **Strains** | |
| --- | --- | --- |
|  | **IS-10** | **BATS-13** |
| Citrate utilization assay | + | + |
| Catalase test | + | + |
| Oxidase test | + | + |
| Indole test | - | - |
| Nitrate reduction test | + | + |

(‘**+**’ implies positive, ‘-’ implies negative)

**Supplementary Table 3:** Quantification of PGP traits of *B. cabrialesii* strains

| **Traits** | **Strains** | |
| --- | --- | --- |
|  | **IS-10** | **BATS-13** |
| Ammonia production (µM/ml) | 3.003 ± 0.27 | 3.58± 0.24 |
| IAA production (µg/ml ) | 09.86 ± 0.65 | 09.39 ± 0.30 |
| Zinc solubilisation (µg/ml) | 36.8 ±0.40 | 29.6 ±0.42 |
| Siderophore production (%) | 68±0.95 | 63.37 ±0.82 |
